# Impact of stereotactic body radiation therapy on systemic therapeutic line change in oligometastatic breast cancer

**DOI:** 10.1016/j.breast.2025.104546

**Published:** 2025-07-21

**Authors:** Julie Leblanc, Alexandre de Nonneville, Camille Nicolas, Anthony Gonçalves, Véronique Favrel, Marguerite Tyran, Morgan Guenole, Leonel Varela, Laurence Gonzague, Agnès Tallet, Claire Petit

**Affiliations:** a Aix-Marseille Univ, CNRS, INSERM, Institut Paoli-Calmettes, Department of Radiotherapy, CRCM, Marseille, France; b Aix-Marseille Univ, CNRS, INSERM, Institut Paoli-Calmettes, Department of Medical Oncology, CRCM, Marseille, France; c Aix-Marseille Univ, CNRS, INSERM, CRCM, Marseille, France

**Keywords:** Oligometastatic, Breast cancer, Stereotactic body radiation therapy (SBRT)

## Abstract

• SBRT is widely used in oligometastatic breast cancer despite limited evidence.
• SBRT delayed systemic therapeutic line change by over 1 year in our cohort.
• Our study may help to identify patients most likely to benefit from SBRT.
• SBRT is a well-tolerated treatment with limited toxicity.
• Randomized trials are needed to assess the benefit of SBRT in terms of survival.

## 1 Introduction

Breast cancer (BC) has been an increasingly common cancer among women worldwide for decades, with currently more than 2,000,000 new cases per year [1,2]. Although it is mostly diagnosed at an early stage, 5–10 % of patients have metastatic disease at the time of diagnosis. Moreover, 20 %–50 % of BC patients will progress to metastatic disease during their lifetimes [3]. This evolution varies according to molecular subtypes, grade, tumor size, lymph node involvement or age. Consequently, many women are living with metastatic breast cancer, which is now recognized as a chronic disease requiring long-term treatment. Recent innovations in systemic therapies such as targeted therapies [4], immunotherapy [5], antibody-drug conjugates [6] or cell cycle inhibitors [7] have significantly improved patient prognosis, allowing longer survival with metastases.

**Fig. 1:**
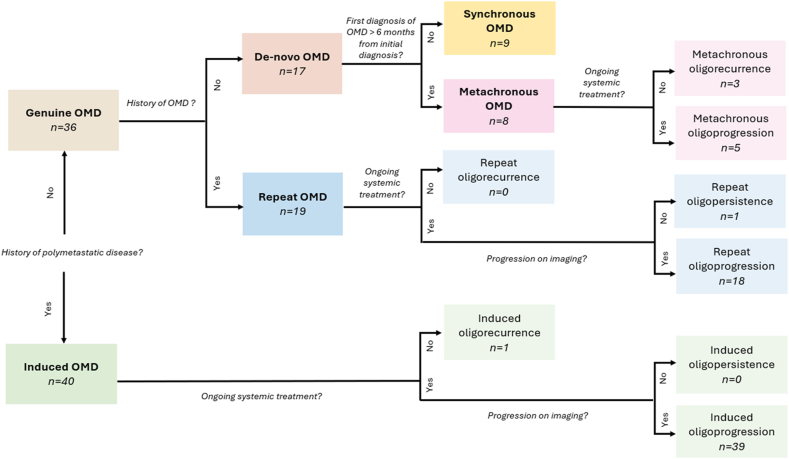
Classification of OMD patients. Abbreviations: OMD = oligometastatic disease.

Among the various metastatic patterns, oligometastatic disease (OMD) is a particular clinical entity. Indeed, Hellman and Weichselbaum described this concept in 1995 as an intermediate state between localized and systemically metastatic disease [8]*.* Thus, it is commonly admitted that OMD is defined by the presence of one to 3–5 metastases [9,10], emphasizing the limited metastatic spread of the disease. However, the number of metastatic sites is not sufficient on its own to characterize accurately OMD and a comprehensive nomenclature for various scenarios is required to better discriminate the variety of situations. With this aim, the European Society for Radiotherapy and Oncology (ESTRO) and the European Organization for Research and Treatment of Cancer (EORTC) recently brought more precisions on OMD [11]. Induced and genuine OMD are distinguished based on prior polymetastatic history. Genuine OMD includes de-novo (no prior OMD) and repeat OMD (prior OMD). De-novo OMD is divided into synchronous (<6 months from initial diagnosis) or metachronous OMD (>6 months). Metachronous, repeat, and induced OMD can be further classified as oligorecurrence, oligoprogression, or oligopersistence, as presented in Fig. 1, adapted from the original classification proposed by Guckenberger et al. [11]. It may be interesting to stratify patients based on their OMD state when multiple OMD states are assessed within the same trial [12]. Moreover, advances in diagnostic imaging and nuclear medicine have facilitated the detection of oligometastases improving the distinction between OMD and polymetastatic disease [13].

Thus, characterization of OMD paved the way to integrate local metastases-directed therapy into the therapeutic arsenal with the aim of achieving long-term local control and improving survival. Traditionally, surgery was the primary modality for removing metastases [14,15], but newer less invasive treatments have recently emerged, such as interventional radiology (radiofrequency, cryoablation, microwave ablation) [16] or stereotactic body radiation therapy (SBRT) [17] which currently play a key role in therapeutic modalities.

Currently, the most compelling evidence supporting the addition of SBRT to standard of care (SOC) in OMD comes from several phase II clinical trials [[18], [19], [20], [21], [22], [23], [24], [25], [26], [27], [28]] and phase III trials including only EGFR positive non-small-cell lung cancer (NSCLC) [29,30]. Regarding breast cancer (BC), there is a lack of evidence showing any benefit from adding SBRT to SOC. Indeed, the phase II/III NRG-BR002 trial showed no benefit to the addition of metastasis directed therapy (SBRT or surgical resection) in PFS [31]. Similarly, *Tsai* et al. [20] compared the addition of SBRT to SOC in oligoprogressive BC and NSCLC, but they only showed PFS benefit of SBRT in NSCLC patients. However, patient characteristics differed when comparing these two studies together, making these results difficult to generalize. Many studies of SBRT in patients with OMD highlighted that patient with BC had a potential for higher survival rate compared to other solid tumors [[32], [33], [34]], particularly among patients with bone metastasis [35,36]. Several single arm prospective trials of SBRT on oligometastatic breast cancer (OMBC) patients corroborate these findings [37,38], achieving a 2-year PFS and OS of 65 % and 100 % respectively in patients with bone-only OMBC [39].

Distant failure after SBRT is one of the major issues in OMD, and treatment decisions in progressive cancer are challenging. To date, conventional treatment for disease progression is changing therapeutic line. In the specific case of oligoprogression, the question arises of changing line or performing a metastasis directed therapy on progressive sites using SBRT, in order to delay the time to subsequent therapy [40]. It may reduce stress, toxicity and improve observance if the current line was previously effective and well tolerated, this allows it to be continued with a generally effective and low-toxicity local treatment. SBRT has been shown in a randomized trial to effectively delay systemic therapy in patients with oligometastatic prostate cancer [21]. However, this benefit has not yet been demonstrated in breast cancer. A phase II study suggests that SBRT may be considered in cases of oligoprogressive luminal breast cancer as an alternative to changing systemic treatment [41].

We conducted a retrospective study to evaluate the impact of SBRT on the time to subsequent systemic treatment (TTST) in patients with OMBC, SBRT-related toxicity and to identify patient or treatment-related factors associated with longer progression-free survival (PFS), overall survival (OS) and local control (LC).

## 2 Materiel & methods

### 2.1 Patient characteristics

We conducted a single-center retrospective study of OMBC patients treated with SBRT on their metastatic lesions at Institut Paoli-Calmettes, Marseille, France, between 2018, January 1st and 2021, December 31st. Inclusion criteria were female patients, aged 18 or older, with a BC regardless of the molecular subtype, presenting with OMD defined by 1–5 metastatic sites at the time of SBRT, and receiving SBRT on at least one site instead of changing systemic therapy which is the standard attitude. They might have received several SBRT courses in case of further progression. Intracranial metastases were accepted. Exclusion criteria were as follows: more than 5 active simultaneous metastases, any change in systemic treatment within six weeks before or after SBRT or the absence of assessment between SBRT and treatment line change and patients who only underwent SBRT at our institution but had their overall oncology management at another institution. Tumor assessment was performed using ^18^FDG-PET CT or body CT scan combined with a bone scan every three months after radiation therapy. In addition, all patients treated for both brain and liver localization had cerebral and hepatic MRI respectively. Phenotype was determined based on the pathological characteristics of metastatic lesions when available; otherwise, the phenotype of primary tumor was used as reference. All patients were classified according the ESTRO/EORTC OMD classification system at the time they received SBRT [11]. The biologically effective dose (BED) was calculated using the linear-quadratic model (α/β = 10 Gy) to compare treatment schemes with clinical outcomes.

### 2.2 Endpoints

The primary endpoint was the time to subsequent therapy (TTST), defined by the time from the end of SBRT to the next systemic treatment. Secondary endpoints were local control (LC), progression-free survival (PFS), overall survival (OS) and toxicity. LC, PFS and OS were respectively defined as the time from end of SBRT to radiological diagnosis of tumor progression within the irradiated volume confirmed by at least two serial imaging, to local or distant progression, and to death from any cause. Toxicity was graded with Common Terminology Criteria for Adverse Events version (CTCAE) 5.0 [42]. The data cut-off was 2024, February 2nd.

### 2.3 Statistical analysis

Descriptive statistics were performed for population and tumor characteristics. Comparisons of categorical and continuous variables were performed using Chi-2 or Fisher's exact tests and Student's T-test, respectively. Time-to-event outcomes were estimated using Kaplan Meier curves and comparisons were done with Log-Rank tests. Optimal cut-off values were determined using the Maxstat method. Univariate analysis was carried out to assess the relationship between patient characteristics, including OMD classification (i.e. synchronous and others), bone metastases, bone-only metastases, brain metastases, number of metastases (1 versus >1), phenotype, estrogen receptor (ER) status, Planning Target Volume (PTV), BED_10_, treatment line (first versus second-line or beyond) and duration of treatment line before SBRT of at least 4 years (This cut-off was chosen based on its optimal discriminative power across variables in our cohort), and the above mentioned endpoints.

For the multivariate analyses, we selected, for each endpoint, the variables that showed the strongest association in the univariate analyses applying a commonly accepted rule of one variable per ten events.

Univariate analyses and multivariate analyses (MVA) were performed using Cox proportional hazards model. All tests were two-sided and p-value <0.05 was considered statistically significant. The Bonferroni correction was applied for multiple comparisons in the post-hoc analysis. All analyses were performed using RStudio software (version February 1, 5019).

### 2.4 Data protection

This study was approved by Institut Paoli-Calmettes internal research steering committee (GSPC). This retrospective study was approved as complying with ethical standards and the 1975 Declaration of Helsinki, as revised in 2013.

## 3 Results

### 3.1 Patients

We identified 246 breast cancer (BC) metastases treated with SBRT between January 2018 and December 2021 at Institut Paoli-Calmettes, Marseille, France. After exclusions of 138 lesions (68 in patients with >5 active metastases, 63 in patients with concurrent systemic therapy change, and 7 in patients with overall oncology management at another institution), 76 patients with 108 metastatic lesions were included in the final analysis **(**Supplementary Fig. S1**)**.

Median follow-up was 42.8 months (IQR: 32.3–53.6 months). The median age at primary diagnosis was 52 years (range: 24–84 years). The median time from primary diagnosis to first metastasis was 30.5 months (IQR: 0.00–70.7), and 28 patients had metastatic disease at diagnosis. In four patients, OMD was diagnosed during adjuvant treatment for localized disease. The median duration of systemic therapy prior to SBRT was 16.8 months (IQR: 8.0–37.0 months) in the overall population. Among the 60 patients who progressed under systemic metastatic treatment, the median duration was 17.5 months (IQR: 8.6–39.0 months). 49 patients (64.5 %) had luminal breast cancer, 13 (17.1 %) had HER2-positive/ER-positive tumors, 4 (5.3 %) had HER2-positive/ER-negative tumors, and 10 (13.6 %) had triple-negative breast cancer.

The median number of metastases per patient at the time of SBRT was 1 (range: 1–5). Fifty-four patients were treated with SBRT on 1 site (71.1 %), thirteen on 2 sites (17.1 %), eight on 3 sites (10.5 %), and one on 4 sites at the same time (1.3 %). Among patients who received SBRT to a single site while presenting with additional metastatic lesions, these sites were either not progressive or managed with interventional radiology or surgery. Only one patient declined SBRT for two progressive lesions. SBRT sites included bone (n = 72, 66.7 %), brain (n = 22, 20.4 %), lung (n = 5, 4.6 %), lymph node (n = 5, 4.6 %), subcutaneous (n = 2, 1.9 %) and liver (n = 2, 1.9 %). Thirty-five patients had bone-only metastases (46.1 %). Among all treated lesions, SBRT was mostly delivered in 3 fractions (n = 66, 61.1 %; range: 1–7) and median BED_10_ was 51.3 Gy (range: 22.5–85.5). Further characteristics of patients and lesions are shown in Table 1, Table 2 respectively.

**Table 1:** Patient characteristics.

		Patients (n = 76)	%
Age, median (min-max)		52 (24–83)	
Initial Tumor stage			
	Tis	1	1.3 %
	T1	12	23.7 %
	T2	32	42.1 %
	T3	12	15.8 %
	T4	9	11.8 %
	NC	4	5.3 %
Initial Nodal stage			
	N0	28	36.8 %
	N1	31	40.7 %
	N2	6	7.9 %
	N3	7	9.2 %
	NC	4	5.3 %
Initial Metastatic stage			
	M0	48	63,2 %
	M1	28	36.8 %
Initial Disease stage			
	1	12	15.8 %
	2	23	30.3 %
	3	11	14.5 %
	4	26	34.2 %
	NC	4	5.3 %
Grade			
	1	4	5.3 %
	2	48	63.2 %
	3	20	26.3 %
	NC	4	5.3 %
Histology			
	Invasive ductal carcinoma	66	86.8 %
	Invasive lobular carcinoma	6	7.9 %
	Other	4	5.3 %
Phenotype			
	Luminal	49	64.5 %
	HER2/ER+	13	17.1 %
	HER2/ER-	4	5.3 %
	TNBC	10	13.6 %
Number of metastases at the time of SBRT			
	1	49	64.5 %
	2	14	18.4 %
	3	7	9.2 %
	4	5	6.6 %
	5	1	1.3 %
Systemic therapy at the time of SBRT			
	Metastatic line	69	90.8 %
	Adjuvant	4	5.3 %
	None	3	3.9 %
Lines of systemic therapy at the time of SBRT			
	Median (mix-max)	1 (0–13)	
Type of systemic therapy at the time of SBRT			
	HT alone	23	30.3 %
	HT + CDK4/6i	25	32.9 %
	HT + CT	1	1.3 %
	HT + HER2 target therapy	4	5.3 %
	HER2 target therapy alone	11	14.5 %
	CT	7	9.2 %
	CT + PARPi	1	1.3 %
	Anti-PDL1	1	1.3 %
	None	3	3.9 %

Abbreviations: CT = chemotherapy, HT = hormone therapy, CDK4/6i = CDK4/6 Inhibitors, PARPi = PARP Inhibitors, SBRT = Stereotactic Body Radiation Therapy, NC = Not Collected.

**Table 2:** Lesion characteristics.

		Number (n = 108)	%
SBRT Sites			
	Bone	72	66.7 %
	Brain	22	20.4 %
	Lung	5	4.6 %
	Lymph node	5	4.6 %
	Liver	2	1.9 %
	Subcutaneous	2	1.9 %
SBRT Schedules			
	18–20 Gy x 1	7	6.5 %
	8–10 Gy x 3	66	61.1 %
	6–8 Gy x 5	29	26.9 %
	Others	6	5.6 %
BED_10_ (Gy)			
	Median (min-max)	51.3 (22.5–85.5)	
PTV (cc)			
	Median (min-max)	16.1 (0.5–139.2)	

Abbreviations: BED = Biologically Effective Dose with α/β = 10 Gy, PTV = Planning Target Volume, SBRT = Stereotactic Body Radiation Therapy, Gy = Gray, cc = cubic centimeter.

We categorized patients and lesions according to ESTRO/EORTC classification for oligometastatic disease (OMD), as shown in Fig. 1. More than half of the patients presented induced OMD (n = 40, 52.6 %) whereas 36 patients were classified as genuine OMD (47.4 %). Among genuine OMD, 19 had repeat OMD and 17 had de-novo OMD. De-novo OMD patients were represented by synchronous in 9 cases (11.8 % of the total population) and metachronous in 8 cases (10.5 % of the total population). Induced oligoprogression was the most frequent state (n = 39, 51.3 % of the total population), followed by repeat oligoprogression (n = 18, 23.7 % of the total population). All patients with synchronous OMD had exclusively bone metastases. Bone-only metastases were significantly associated with synchronous status (p < 0.01) and tended to be associated with positive ER (p = 0.08). Brain metastases were associated with HER2 status (p < 0.01).

### 3.2 Primary endpoint: time to subsequent therapy (TTST)

**Fig. 2:**
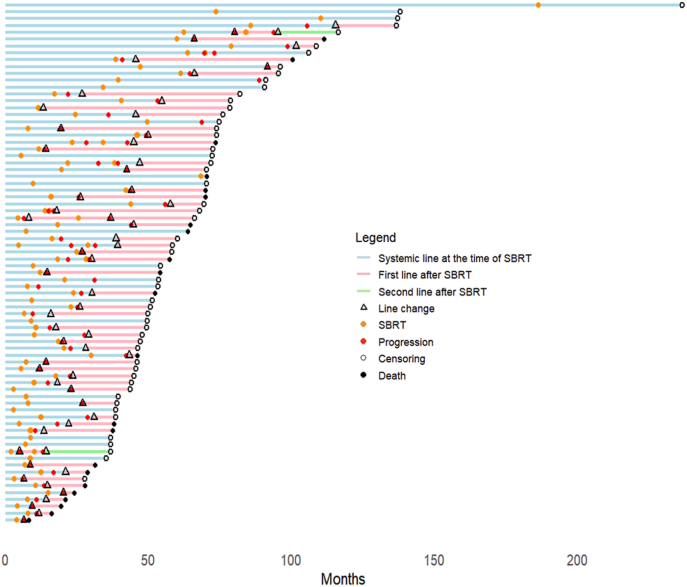
Swimmer plot illustrating treatment line history and disease progression. Time “0” is corresponding to the initiation of the treatment line the patient was receiving at the time of SBRT. SBRT = Stereotactic Body Radiation Therapy.

**Fig. 3:**
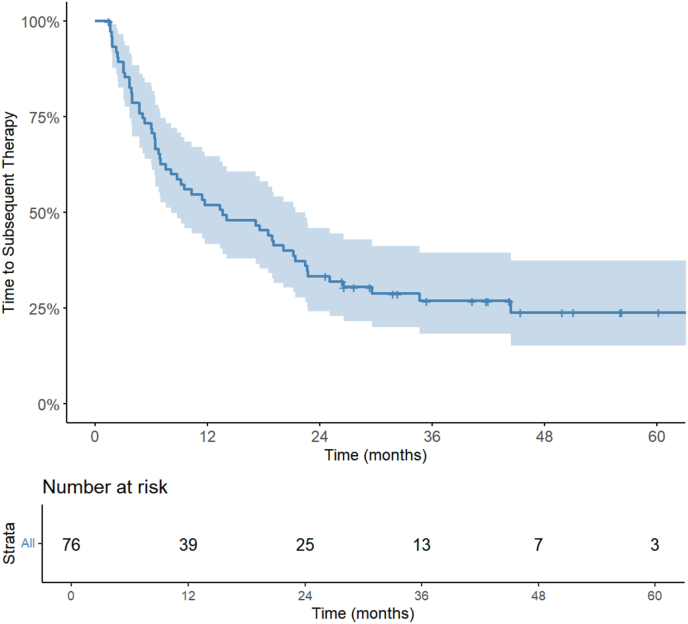
Kaplan–Meier curve of TTST in overall population.

A swimmer plot illustrating treatment line history and disease progression is presented in Fig. 2. Median TTST was 13.7 months (95 %CI: 8.2–22.5 months). During the follow-up period, 72.3 % of patients (55/76) patients had a change in systemic treatment. Forty patients of 76 were still receiving the same treatment at 1 year (52.6 %) and 26 patients at 2 years (34.2 %). Kaplan-Meier curve for TTST is presented in Fig. 3. In univariate analysis, synchronous disease, treatment duration of at least 4 years prior to SBRT and first line treatment were associated with longer TTST **(**Supplementary Fig. S2**)**. In multivariate analysis, synchronous disease and treatment duration of at least 4 years prior to SBRT remained significant, and brain metastases appeared also associated with longer TTST, as shown in Table 3. No other variables were found to be associated with TTST.

**Table 3:** Uni- and multivariate analysis in overall population.

Univariate analysis								
	TTST		PFS		OS		LC	
		***p-value***		***p-value***		***p-value***		***p-value***
Synchronous		**<0.01**		**<0.01**		0.11		0.06
Duration of line prior to SBRT >4 years		**0.03**		0.08		0.41		0.32
First line		**0.02**		**<0.01**		**<0.01**		0.34
Brain metastases		0.11		0.78		0.72		0.08
Bone-only metastases		0.26		**0.04**		0.68		**<0.01**
Bone metastases		0.49		0.53		0.44		0.23
ER-positivity		0.63		0.09		0.02		0.82
Number of metastases (1 vs > 1)		0.37		0.06		0.42		0.37
BED >55 Gy		0.22		0.73		0.84		**0.03**
BED >44 Gy		0.54		0.93		**0.045**		0.41
PTV 10.2 cc		0.43		**0.03**		0.59		**0.02**

Multivariate analysis								
	**TTST** **HR (95 % CI)**	***p-value***	**PFS** **HR (95 % CI)**	***p-value***	**OS HR (95 % CI)**	***p-value***	**LC HR (95 % CI)**	***p-value***

Synchronous	0.13 (0.03–0.63)	**0.01**	0.05 (0.006–0.40)	**0.01**				
Duration of line prior to SBRT >4 years	0.29 (0.1–0.83)	**0.02**	0.35 (0.14–0.88)	**0.03**				
First line	0.82 (0.45–1.51)	0.53	0.66 (0.37–1.18)	0.16	0.18 (0.06–0.54)	**0.02**		
Brain metastases	0.33 (0.12–0.89)	**0.03**						
Bone-only metastases	0.86 (0.47–1.65)	0.70	1.16 (0.62–2.2)	0.60			0.28 (0.09–0.89)	**0.03**
ER-positivity			0.69 (0.32–1.5)	0.35	0.70 (0.22–2.22)	0.55		
BED >55 Gy							0.13 (0.017–0.96)	**0.045**
BED >44 Gy					0.43 (0.16–1.14)	0.09		
PTV 10.2 cc			1.0 (0.55–1.84)	0.99			1.87 (0.75–4.6)	0.18

Abbreviations: BED = Biologically Effective Dose with α/β = 10 Gy, PTV = Planning Target Volume, SBRT = Stereotactic Body Radiation Therapy, Gy = Gray, cc = cubic centimeter.

### 3.3 Secondary endpoints

#### 3.3.1 Progression-free survival (PFS) and Ovarall survival (OS)

Median PFS was 9.8 months (95 %CI: 4.9–17.4 months). One-year and 2-year PFS were respectively 42.7 % (95 %CI: 32.8–55.5 %) and 25.3 % (95 %CI: 17.2–37.4 %). At the end of follow-up, disease progression was observed in 59 patients (77.6 %), with 38 experiencing distant progression (64.4 %), 20 both distant and local progression (33.9 %), and one local progression limited to the SBRT site (1.7 %). Factors associated with longer PFS in univariate analysis were synchronous disease (p < 0.001), first line treatment (p = 0.03), having bone-only metastases (p = 0.04) and a PTV larger than 10.2 cc (p = 0.03). A trend toward better PFS was observed in patients with a single metastasis compared to multiple (p = 0.06), in ER-positive patients (p = 0.09), and in those who had received systemic therapy for more than 4 years prior to SBRT (p = 0.08) (Supplementary Figures S3). In MVA, synchronous presentation and treatment duration >4 years prior to SBRT were associated with longer PFS as shown in Table 3.

**Fig. 4:**
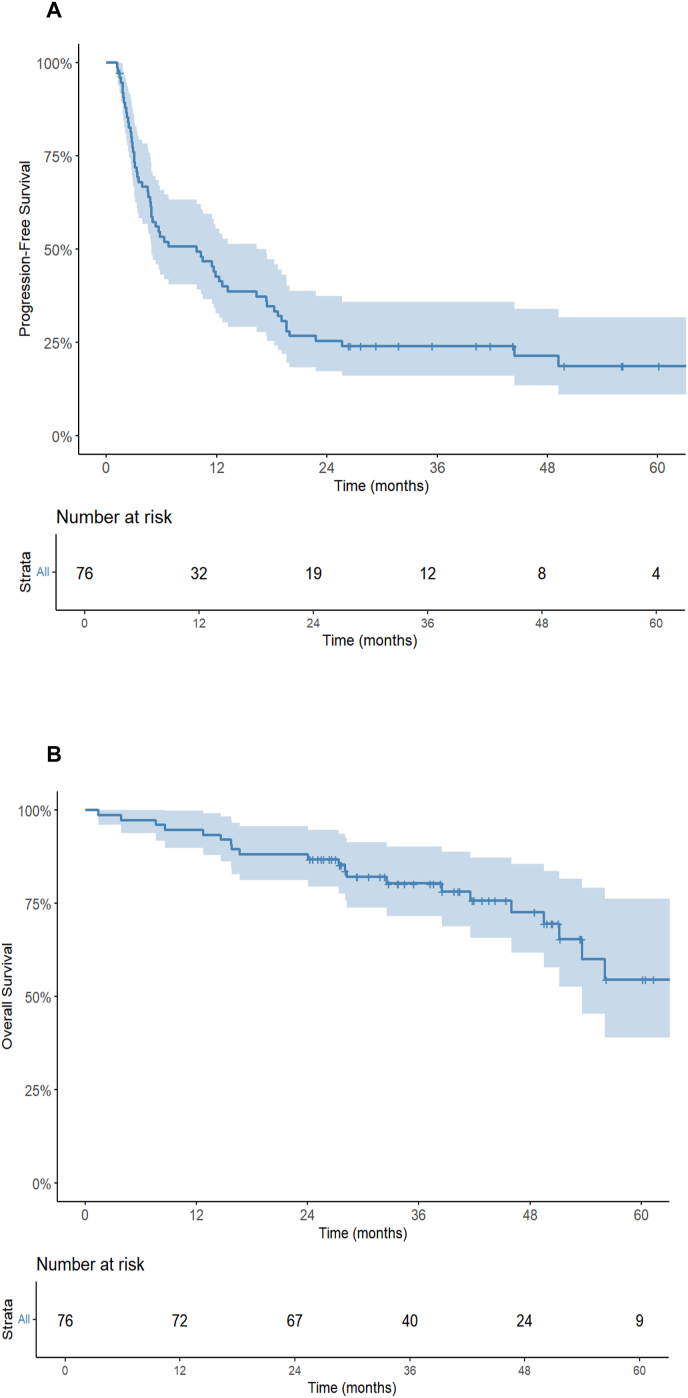
Kaplan–Meier curves of PFS **(A)** and OS **(B)** in overall population.

Regarding OS, the median was not reached (95 %CI: 53.6-not reached). Kaplan-Meier curves of PFS and OS are shown in Fig. 4. One-year and 2-year OS were 94.7 % (IC95 %: 89.8–99.9 %) and 88.3 % (IC95 %: 82.2–95.7 %), respectively. Twenty-one deaths (27.6 %) occurred during the follow-up period. Factors associated with better OS in univariate analysis were first line treatment, positive ER-status and a BED higher than 44 Gy (Supplementary Figures S4). In MVA, first line treatment was the only factor associated with better OS (HR = 0.18, 95 %CI: 0.06–0.54, p = 0.02), as presented in Table 3.

#### 3.3.2 Local control (LC)

Twenty-one lesions were not controlled by SBRT (19.4 %) at the end of follow-up, corresponding to a local control rate of 90.5 % at 1 year (95 % CI: 85.0–96.3 %) and 84.3 % at 2 years (95 % CI: 77.5–91.7 %). Improved LC was associated with bone-only metastases (p = 0.01), a BED greater than 55 Gy (p = 0.03) and a PTV larger than 10.2 cc (p = 0.02) (Supplementary Fig. S5). In MVA, bone-only metastases and BED remained significantly associated with improved LC, as illustrated in Table 3.

#### 3.3.3 Toxicity

Three cases of brain radionecrosis occurred in a single patient (one grade 1 and two grade 3). Two out of three were treated surgically. No other relevant toxicities were observed.

### 3.4 Subgroup analysis

#### 3.4.1 Oligometastatic disease (OMD) classification

The EORTC-ESTRO classification of OMD is illustrated in Fig. 1. Median TTST and median PFS were not reached in patients with de-novo OMD (i.e. synchronous or metachronous disease). TTST, PFS and LC were significantly longer in de-novo OMD compared to repeat or induced OMD (p = 0.03, p < 0.01 and p = 0.03, respectively). Post-hoc analysis showed that TTST, PFS and LC were significantly better in case of de-novo compared to induced OMD, but not significantly different from repeat OMD. The benefit observed in de-novo OMD in both TTST and PFS was mainly driven by patients with synchronous OMD (p = 0.02 and p < 0.01, respectively). Metachronous, repeat and induced OMD were not significantly different in TTST and PFS. Overall survival did not differ significantly between de-novo, repeat and induced OMD (p = 0.08) (Supplementary Fig. S6).

#### 3.4.2 Oligoprogressive patients receiving systemic therapy at the time of SBRT

Among these 60 patients, median TTST was 11.8 months (95 %CI: 7.0–21.2 months); 31 patients of 76 were still receiving the same treatment at 1 year (40.8 %) and 18 patients at 2 years (30.0 %). Median PFS was 5.8 months (95 %CI: 4.8–12.3 months) and median OS was not reached (95 %CI: 51.2-not reached). Local control rate was 85.8 %. In multivariate analysis, treatment duration >4 years before SBRT is associated with longer TTST and PFS (p = 0.01 and p = 0.03, respectively). Having SBRT during the first line of systemic therapy (p = 0.01) was the only factor associated with an improved OS in MVA (p = 0.01). No factor was associated with better LC.

#### 3.4.3 Exclusion of brain metastases

Median TTST was 11.6 months (95 %CI: 7.0–20.1 months), median PFS was 8.3 months (95 %CI: 4.9–17.4 months) and median OS was not reached (95 %CI: 51.2-not reached). Local control rate was 83.7 %. In multivariate analysis, both synchronous presentation and treatment duration >4 years before SBRT are associated with longer TTST and PFS. Having SBRT during the first line of systemic therapy (p < 0.01) was associated with improved OS and having bone-only metastases was associated with better LC (p = 0.01).

## 4 Discussion

This retrospective single-institution study is among the largest investigating the impact of SBRT in OMBC patients and the first to include intracranial metastases. We reported a median time to subsequent systemic therapy (TTST) of 13.7 months in overall population and 11.8 months in oligoprogressive patients, with more than half of patients remaining on the same systemic regimen at one year post-SBRT. Our findings suggest that SBRT may allow selected OMBC patients to maintain an effective systemic therapy beyond conventional progression thresholds, with excellent tolerability. Importantly, we identified synchronous OMD, a prior systemic therapy duration of at least four years and brain metastases as independent predictors of longer TTST. While this observation does not imply causality in the absence of a control arm, it supports the hypothesis that local treatment with SBRT may contribute to delaying systemic escalation in appropriately selected patients. However, it is important to note that synchronous patients all received SBRT as upfront treatment, making it challenging to determine the specific role of SBRT in delaying the time to subsequent therapy. Nonetheless, survival outcomes in this population are comparable to those of certain patients with locally advanced disease, suggesting that a curative approach using SBRT to target metastatic sites may be of interest. Longer 10.13039/100015142PFS observed in our cohort supports the hypothesis that this state may represent a distinct clinical entity warranting further investigation. This question is currently being addressed in the STEREO-SEIN (NCT02089100) and TAORMINA (NCT05377047) trials [55] and our work provides retrospective data in this setting while awaiting more definitive results from these studies. Moreover, none of the patients received Trastuzumab-Deruxtecan, which, considering the intracranial efficacy of this treatment [43], may influence the interpretation of our results regarding this subgroup of patients with brain metastases.

Several retrospective studies evaluated the impact of SBRT on TTST in the context of OMBC. *Wijetunga* et al. [44] reported a median TTST of 28 months, which is twice as long as in our study. This difference may be explained by the fact that the population included was different: almost half of their patients had de-novo OMD, whereas induced OMD patients were mainly included in our study. *Nicosia* et al. [45] reported for their part a median TTST of 8 months. They included 79 cases of oligoprogressive breast cancer, mostly in ER-positive patients (86 %), with bone lesions representing 35 %. They identified the number of oligometastases (single versus multiple) as the only factor associated with a longer TTST. Similarly, *Weykamp* et al. [46] and *Milano* et al. [47] have identified having a single localization as a predictive factor of PFS in OMBC, while it is only suggestive in PFS in our cohort.

Furthermore, having bone-only metastases appears to ensure favourable survival outcomes. In our cohort, it was associated with better PFS only in univariate analysis. *Nagpal* et al. [48] found the presence of metastases outside the bone was associated with poorer PFS and OS compared to bone-only disease. A long-term follow up of prospective study from *Milano* et al. [36] reported that bone-only metastases were associated with younger age, synchronous disease, and ER-positivity. OS was significantly higher in patients with bone-only metastases, with 75 % (9 out of 12) surviving over ten years. Moreover, the higher local control rate in bone-only metastases in our study may suggest a potentially distinct biological behaviour. Thus, bone-only metastases may represent a distinct clinical entity. A study on single-fraction radiation therapy in patients with bone-only metastases from OMBC [39] involving 15 patients across all types of OMD (synchronous, repeat, and induced) and with a majority luminal phenotype, observed a 2-year local control and PFS rate of 100 % and 67 % respectively, without reported deaths. Notably, 72 % of luminal patients continued the same endocrine therapy after SBRT. Likewise, the phase II AVATAR trial assessed the continuation of CDK 4/6 inhibitors plus aromatase inhibitors in 32 oligoprogressive luminal BC patients undergoing SBRT among which 71 % had bone-only metastases. They reported a median TTST of 10.4 months, with 46 % of patients maintaining treatment at 12 months and 33 % progressions suitable for a second course of SBRT for oligoprogression to further delay systemic therapy change [49]. This highlights the potential role of SBRT in delaying the change of systemic therapy in selected patients.

Beyond disease-related factors, SBRT patterns also have an impact on survival. In our cohort, a BED higher than 44 Gy was associated with improved OS in UVA. Consistently, *Nicosia* et al. [45] reported that a BED greater than 70 Gy was correlated with better OS in patients with OMBC in multivariate analysis. Several studies confirmed this association between BED and OS improvement in patients treated with SBRT for liver metastases from colorectal cancer [50,51]. Surprisingly, we found that a smaller PTV was associated with worse PFS and LC. However, in our cohort, PTVs were smaller in brain lesions, and larger in bone lesions of bone-only patients. In the subgroup analysis excluding brain metastases, PTV size was no longer associated with poorer outcomes.

Several studies reported that a longer metastasis-free interval was associated with better survival outcomes. *Deluche* et al. [52] reported worse OS when the interval was less than 24 months, while *Scorsetti* et al. [37] observed better outcomes with an interval exceeding 12 months. These results are consistent with our observation that a long duration of systemic therapy before SBRT correlates with better TTST and PFS, both in the overall population and in the subgroup excluding synchronous patients.

Based on a comprehensive analysis of the literature, *Colciago* et al. [53] identified factors likely associated with better survival outcomes following SBRT in OMBC patients, including the presence of a single lesion, bone-only metastases, ER-positivity, a metastasis-free interval longer than 24 months, smaller PTV and good performance status.

However, two prospective randomized trials did not support the use of SBRT to standard of care (SOC) in OMBC. The phase II/III NRG-BR002 trial showed no benefit in PFS to the addition of metastasis directed therapy (SBRT or surgical resection) [31]. Patients had up to 4 extra-cranial metastases (n = 1 for 60 %), were mostly ER-positive (80 %), metachronous (79 %) and had to be receiving their first-line systemic therapy for no longer than 12 months. Moreover, *Tsai* et al. [20] compared the addition of SBRT to SOC in both oligoprogressive BC and non-small cell lung cancer (NSCLC) but they only showed a benefit in PFS of adding SBRT for NSCLC patients. Notably, more than half of BC patients initially presented with more than five metastatic sites, one-third had triple-negative disease, and the median number of prior systemic therapies was 3, indicating an advanced disease. Differences in patient characteristics across studies make direct comparisons challenging and reduce the generalizability of the results. Recently, STOP trial [54] compared SOC versus SBRT in 90 patients with oligoprogressive cancer and surprisingly found a benefit in PFS for breast cancer subgroup treated with SBRT. However, the small sample size (n = 12) and the lack of detailed patient characteristics limit the interpretation of these findings. These findings highlight the need for further investigation in OMBC.

Several ongoing randomized trials aim to assess PFS in OMBC patient treated by SBRT. Phase III trials STEREO-SEIN (NCT02089100) and TAORMINA (NCT05377047) [55] aim to evaluate the role of metastasis-directed SBRT by comparing SBRT versus SOC as a first-line treatment for de novo OMBC. OLIGOMA study [56] include patients with de-novo, oligoprogressive, or oligopersistent OMBC classified by ESTRO criteria, and those with brain metastases. Both trials evaluate PFS as their primary endpoint.

Our study has strengths and limitations. The use of the ESTRO/EORTC classification may ensure better reproducibility and comparability with future studies, providing a standardized framework for categorizing OMD. Additionally, this is the first study to include brain metastases in this context, broadening the applicability of SBRT across different metastatic sites. Focusing on time to subsequent therapy is a clinically relevant endpoint reflecting treatment sequencing strategy in OMBC, with real-life implications. We identified some factors associated with improved outcomes in terms of TTST, PFS, OS and LC, which are consistent with previously published data. However, our work is a retrospective single-institution study that may lead to selection bias and confounding factors. The absence of a control arm prevents formal evaluation of SBRT's causal impact on delaying systemic therapy. In retrospective studies, constructing a meaningful comparator group (i.e., OMBC patients not treated with SBRT but otherwise eligible) is extremely challenging and subject to substantial confounding. For instance, patients who did not receive SBRT may have had more aggressive disease, poorer response to systemic therapy, or contraindications to local treatment, introducing major bias. Finally, the study population was biologically and therapeutically heterogeneous. Patients presented with diverse breast cancer subtypes (luminal, HER2-positive, triple negative), and were managed with various systemic regimens, including endocrine therapy, CDK4/6 inhibitors chemotherapy, anti-HER2 agents or immune checkpoint inhibitor. These differences likely influenced both SBRT indications and subsequent treatment strategies, introducing variability in outcomes across subgroups. While this heterogeneity reflects real-world clinical practice, it complicates the interpretation of treatment effects. To mitigate this limitation, multivariate analyses were performed to adjust for key prognostic factors, including ER status, number and location of metastases, prior treatment duration, number of treatment line, and radiation parameters. Although the cohort is relatively large, the small sample size for some subgroups (e.g., synchronous OMBC) limits generalizability. All previously described elements underscore the need for larger cohorts or multiple dedicated randomized trials or multiple dedicated randomized controlled trials involving selected patient populations according to the most significant risk factors.

## 5 Conclusion

Our results suggest that SBRT may be safely used to defer systemic therapy changes in selected OMBC patients, particularly those with synchronous OMD, long prior treatment duration or in case of brain metastases. However, this question requires further validation through prospective and randomized trials to better define which OMBC patient benefit from SBRT.

## CRediT authorship contribution statement

**Julie Leblanc:** Writing – review & editing, Writing – original draft, Visualization, Validation, Investigation, Formal analysis, Data curation. **Alexandre de Nonneville:** Writing – review & editing. **Camille Nicolas:** Writing – review & editing. **Anthony Gonçalves:** Writing – review & editing. **Véronique Favrel:** Writing – review & editing. **Marguerite Tyran:** Writing – review & editing. **Morgan Guenole:** Writing – review & editing. **Leonel Varela:** Writing – review & editing. **Laurence Gonzague:** Writing – review & editing. **Agnès Tallet:** Writing – review & editing, Conceptualization. **Claire Petit:** Writing – review & editing, Validation, Supervision, Methodology, Formal analysis, Conceptualization.

## Declaration of competing interests

AdN: Gilead (consulting fees, lecture fees, congress invitations), Daiichi Sankyo (consulting fees, lecture fees, congress invitations), Astra Zeneca (consulting fees, lecture fees, congress invitations), Seagen (consulting fees), Lilly (consulting fees, lecture fees, congress invitations), Novartis (consulting fees, lecture fees, congress invitations), MSD (consulting fees, congress invitations, lecture fees), Pfizer (research grants paid to institution, lecture fees, congress invitations), Promise Proteomics (consulting fees).

CP: Astra Zeneca (congress invitation), Elekta (travel grant), Pfizer (lecture fees paid to institution).

JL, AG, AT, CN, VF, MT, MG, LV, LG: no COI.
