# Supplementary material for: Impact of stereotactic body radiation therapy on systemic therapeutic line change in oligometastatic breast cancer
Source: Breast. 2025 Jul 21;83:104546. doi: 10.1016/j.breast.2025.104546 (PMC12329079; doi:10.1016/j.breast.2025.104546)
Supplement: Multimedia component 1 [file mmc1.docx]

Identification of 246 breast cancer metastases treated with SBRT

Exclusion of 138 lesions:

- 68 in patients with > 5 active metastases
- 63 in patients with concurrent systemic therapy change
- 7 in patients with overall oncology management at another institution

Inclusion of 76 patients:

- 54 recieving SBRT on 1 site
- 13 recieving SBRT on 2 sites
- 8 recieving SBRT on 3 sites
- 1 recieving SBRT on 4 sites`

Inclusion of 108 metastases treated with SBRT

Abbreviations: SBRT = Stereotactic Body Radiation Therapy

**Supplementary Figures S1.** CONSORT diagram of patient and lesion inclusions

**A**
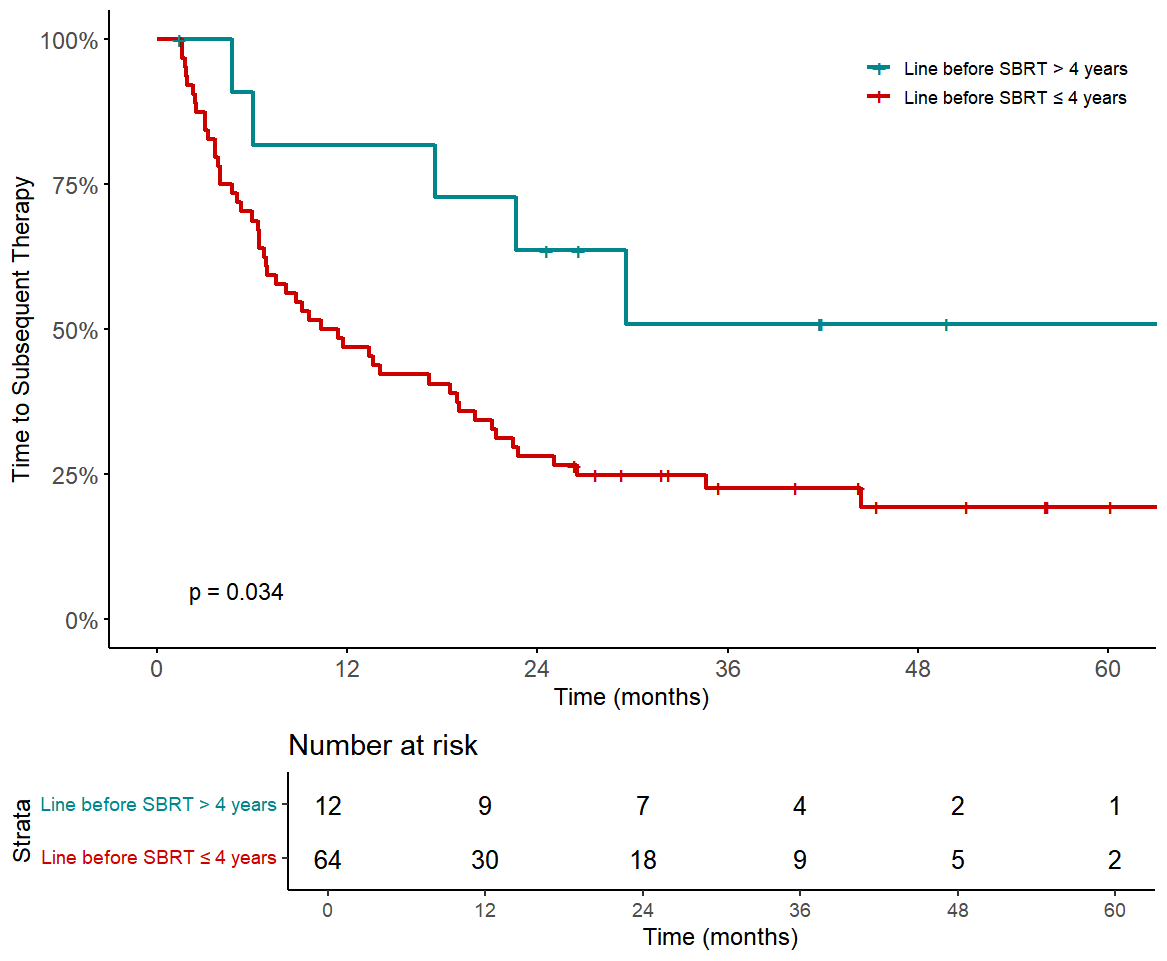


**B**

**
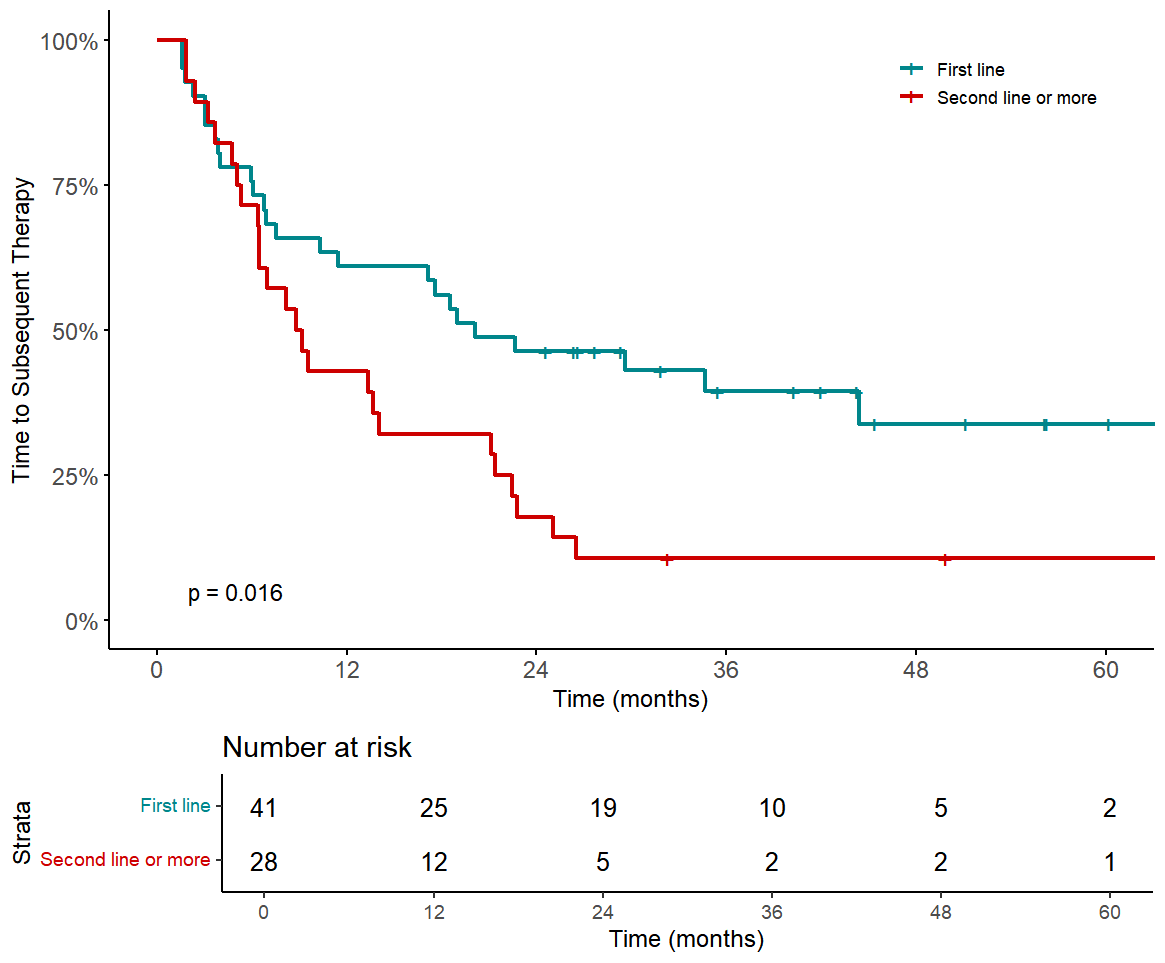
**

**C**

**
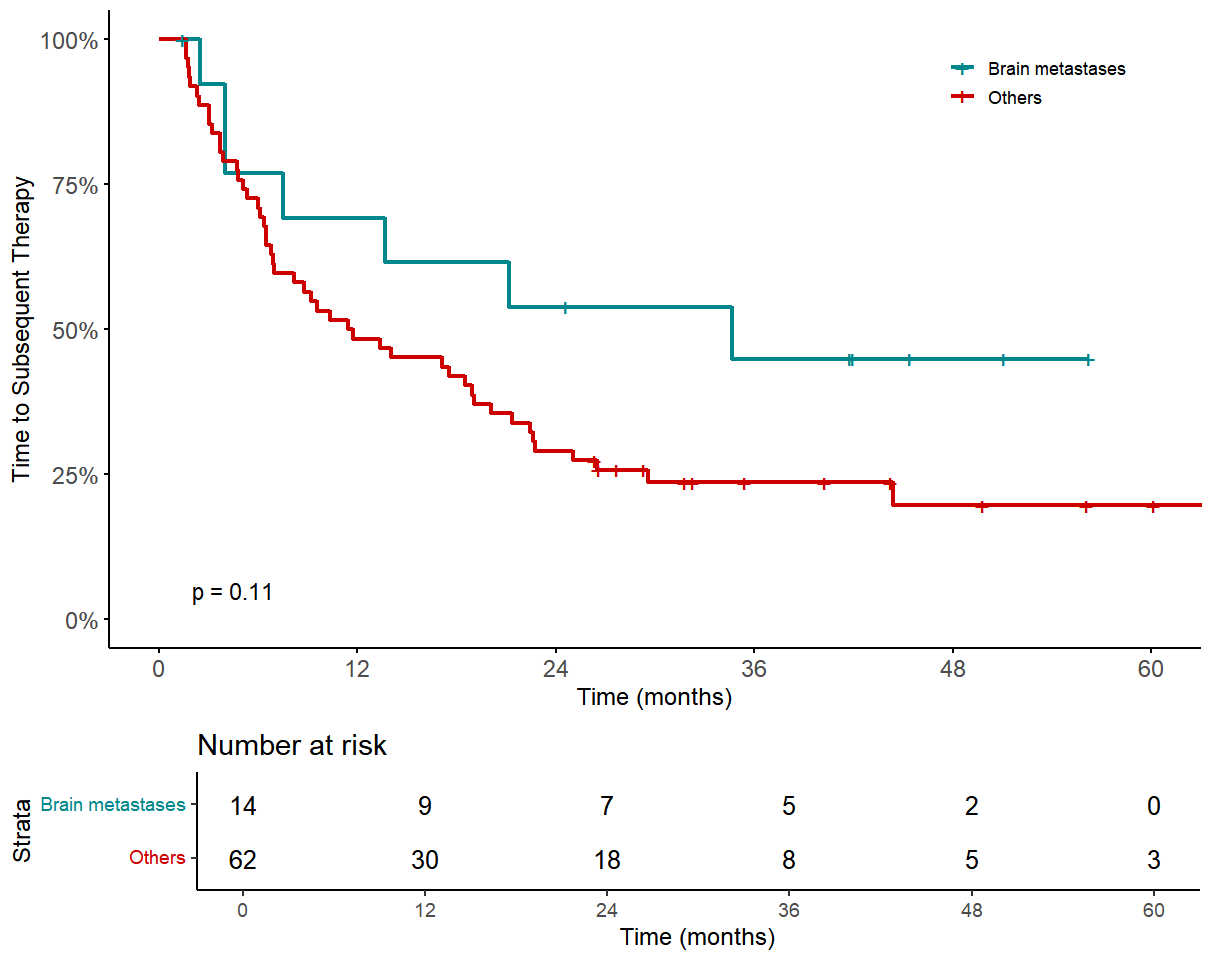
**

**Supplementary Figure S2**. Kaplan-Meier curves of TTST according to duration of treatment line before SBRT (cut-off: 4 years) **(A),** number of current line (first versus second or more) **(B)** and brain metastases **(C)**


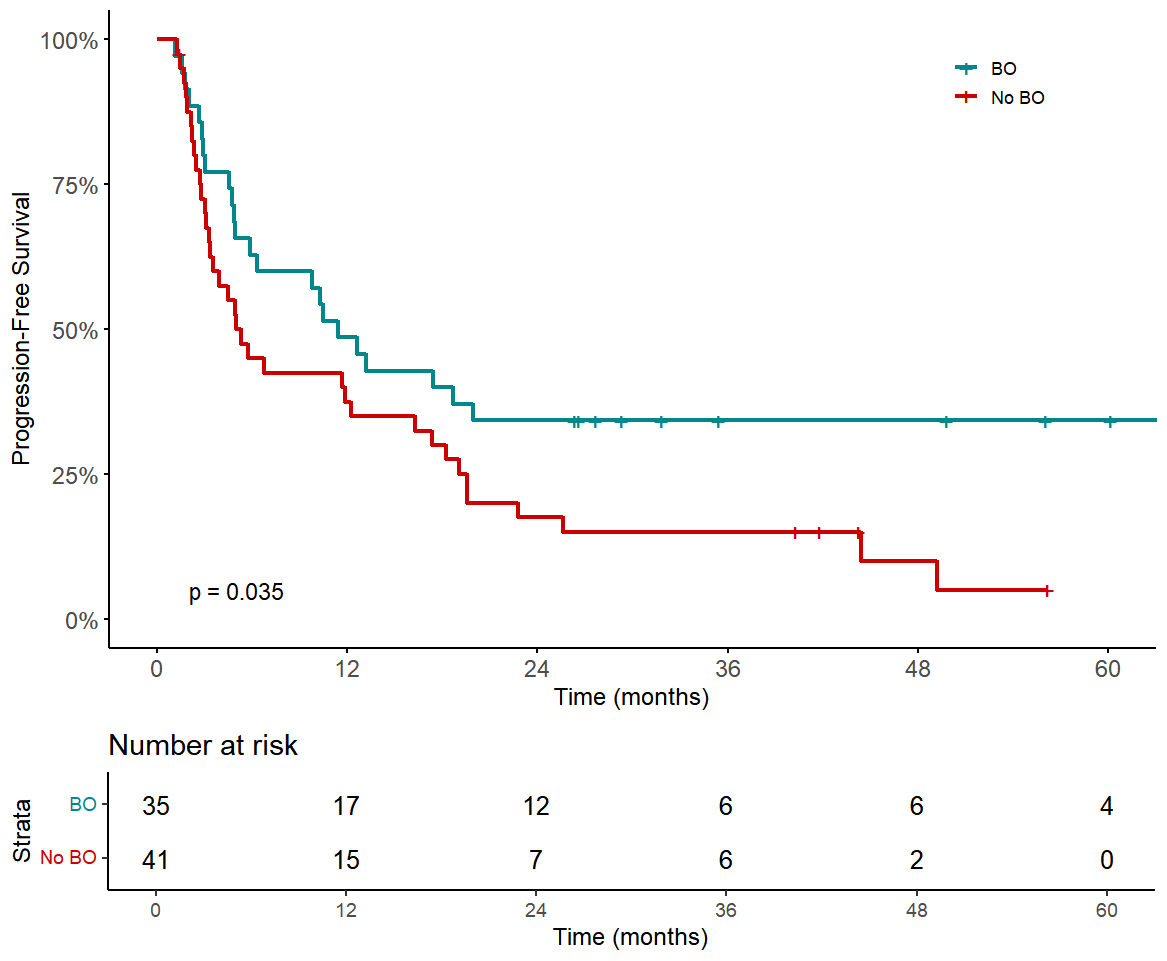
**A**


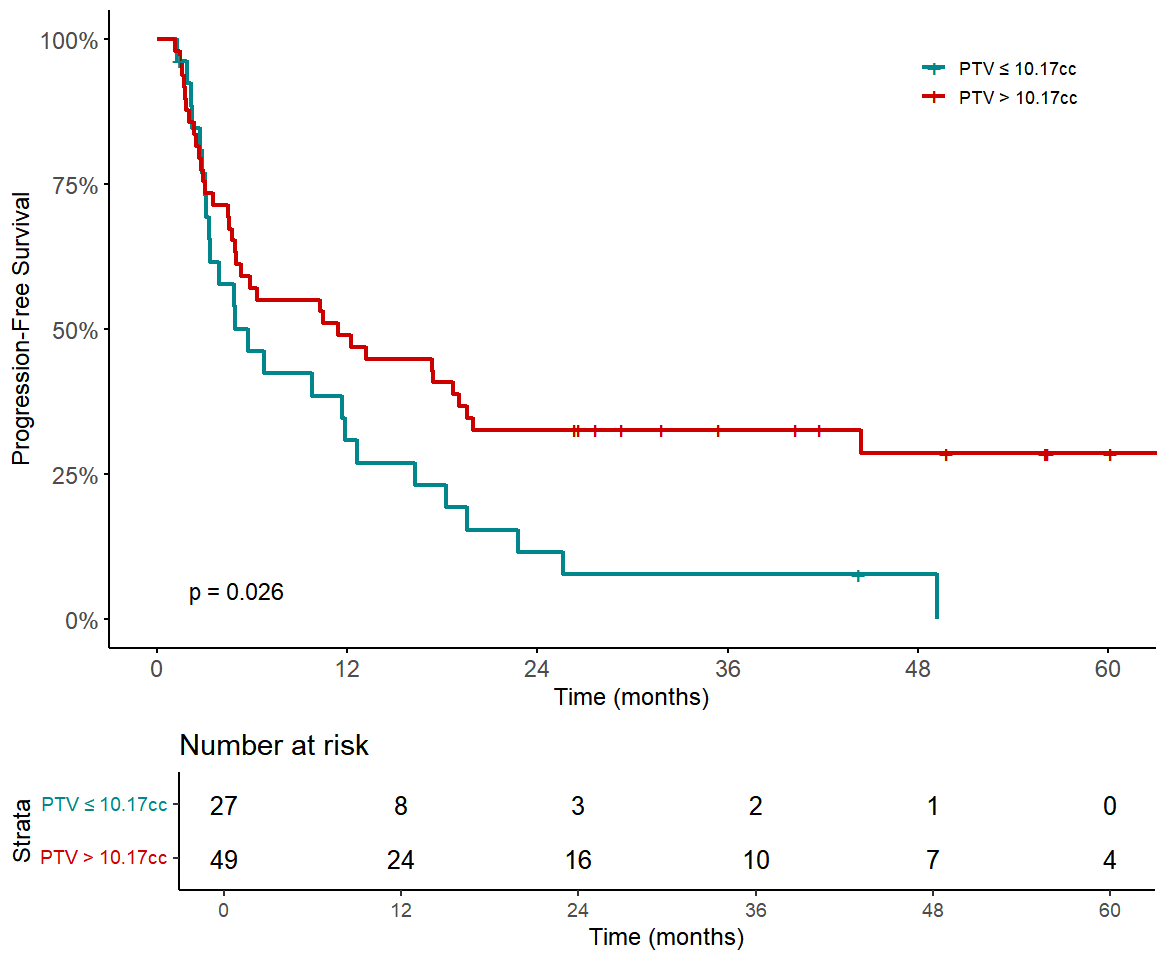
**B**

**C**


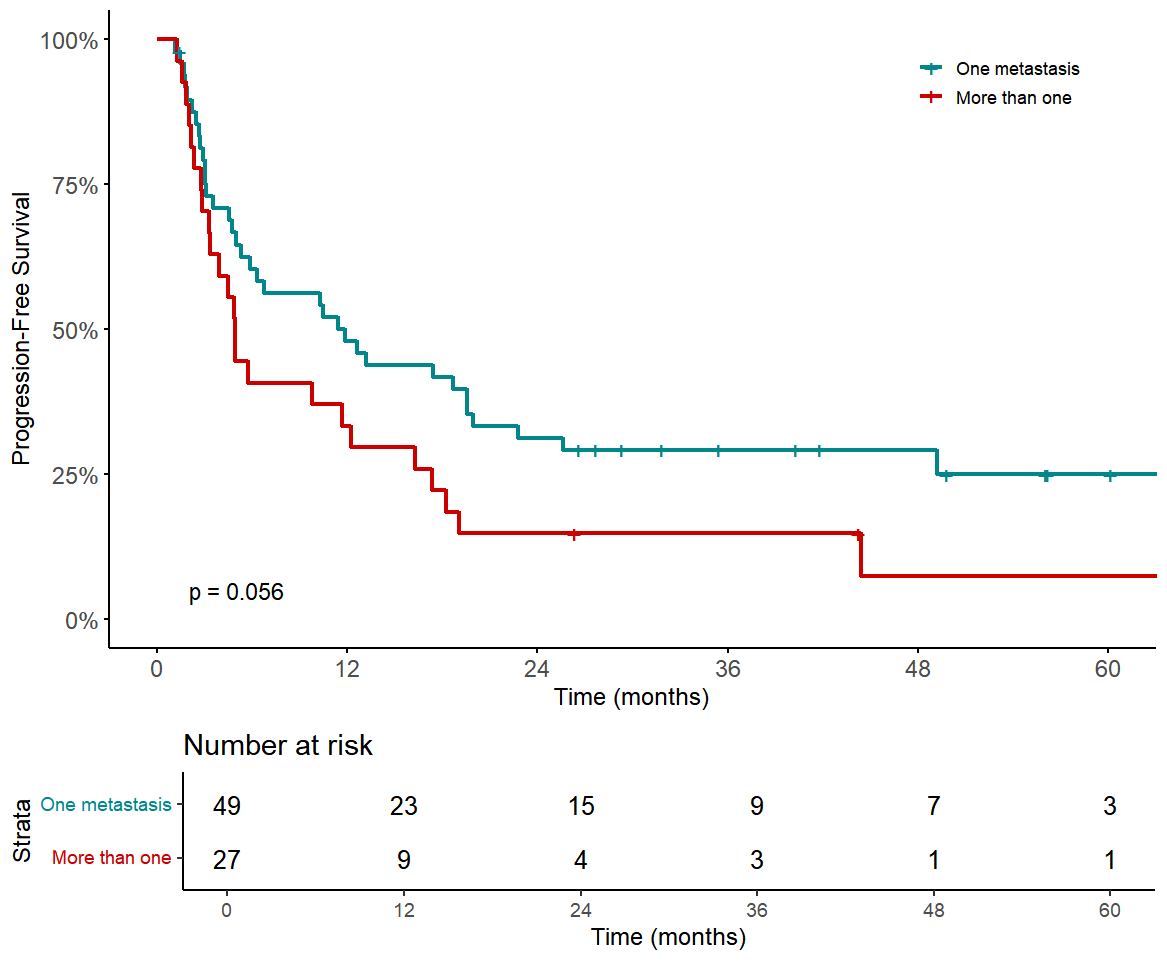


**D**

**
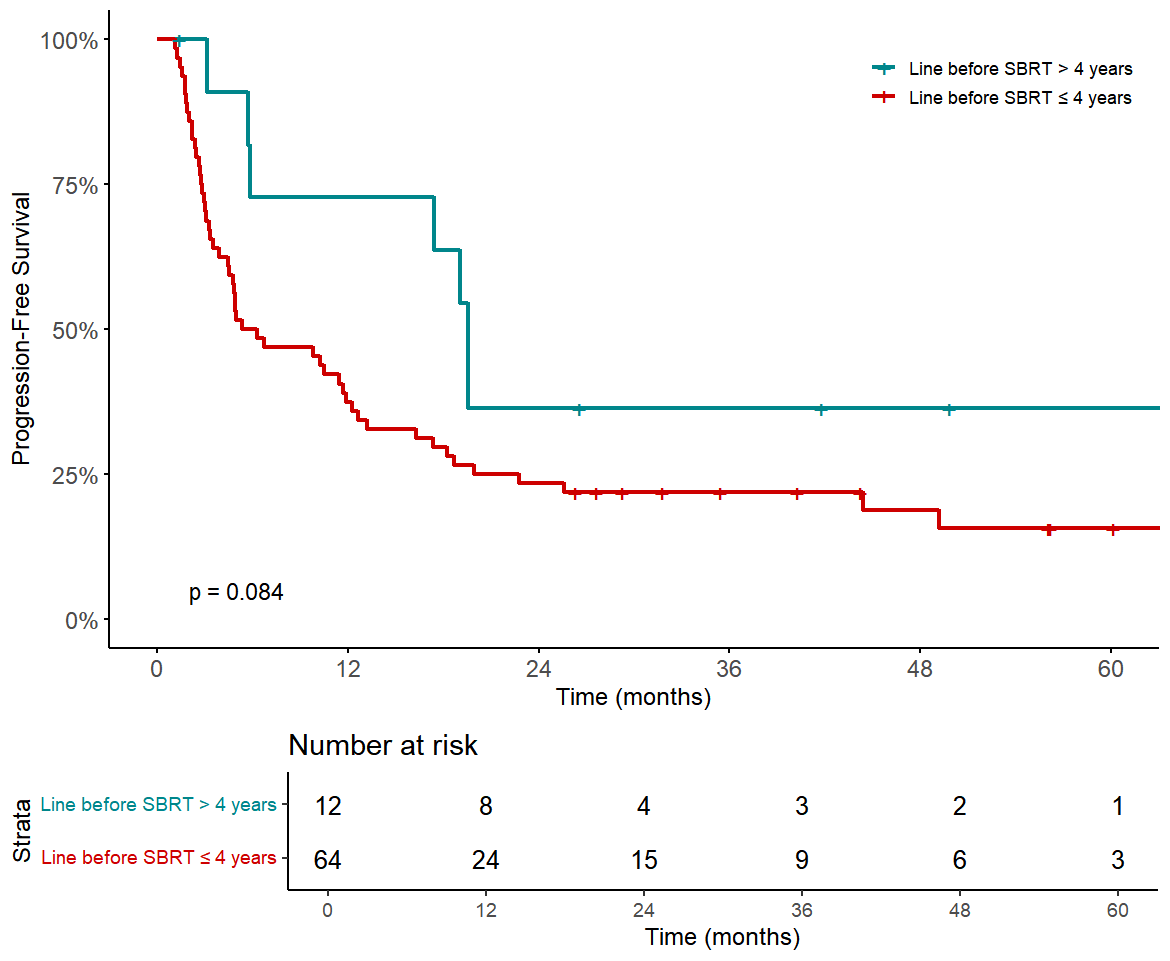
**

**E**

**
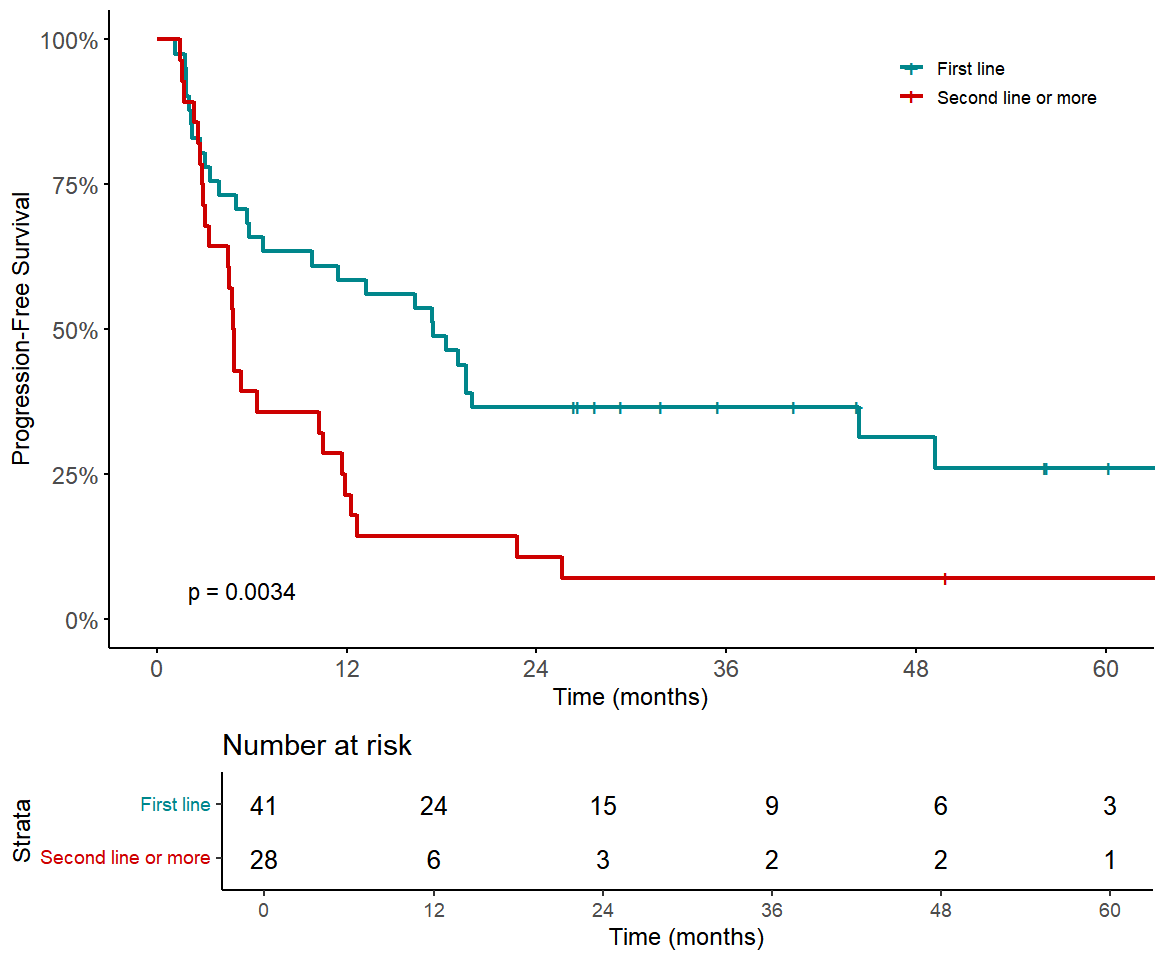
**

**
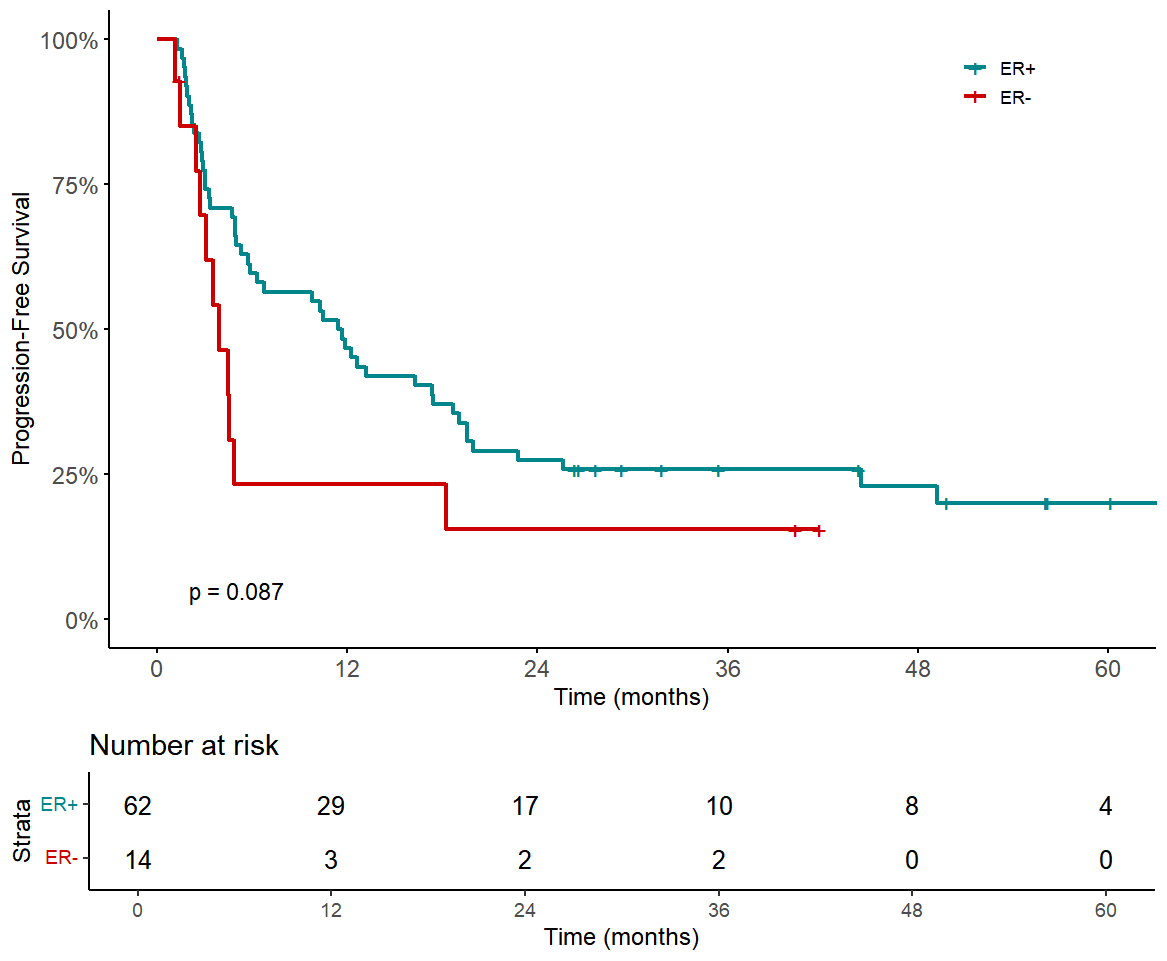
F**

**Supplementary Figures S3**. Kaplan-Meier curves of PFS according to bone-only metastases status (**A**), PTV size (cut-off: 10.17cc) (**B**), number of metastases (**C**), duration of treatment line before SBRT (cut-off: 4 years) (**D**), number of current line (first versus second or more) **(E)** and ER-status (**F**)


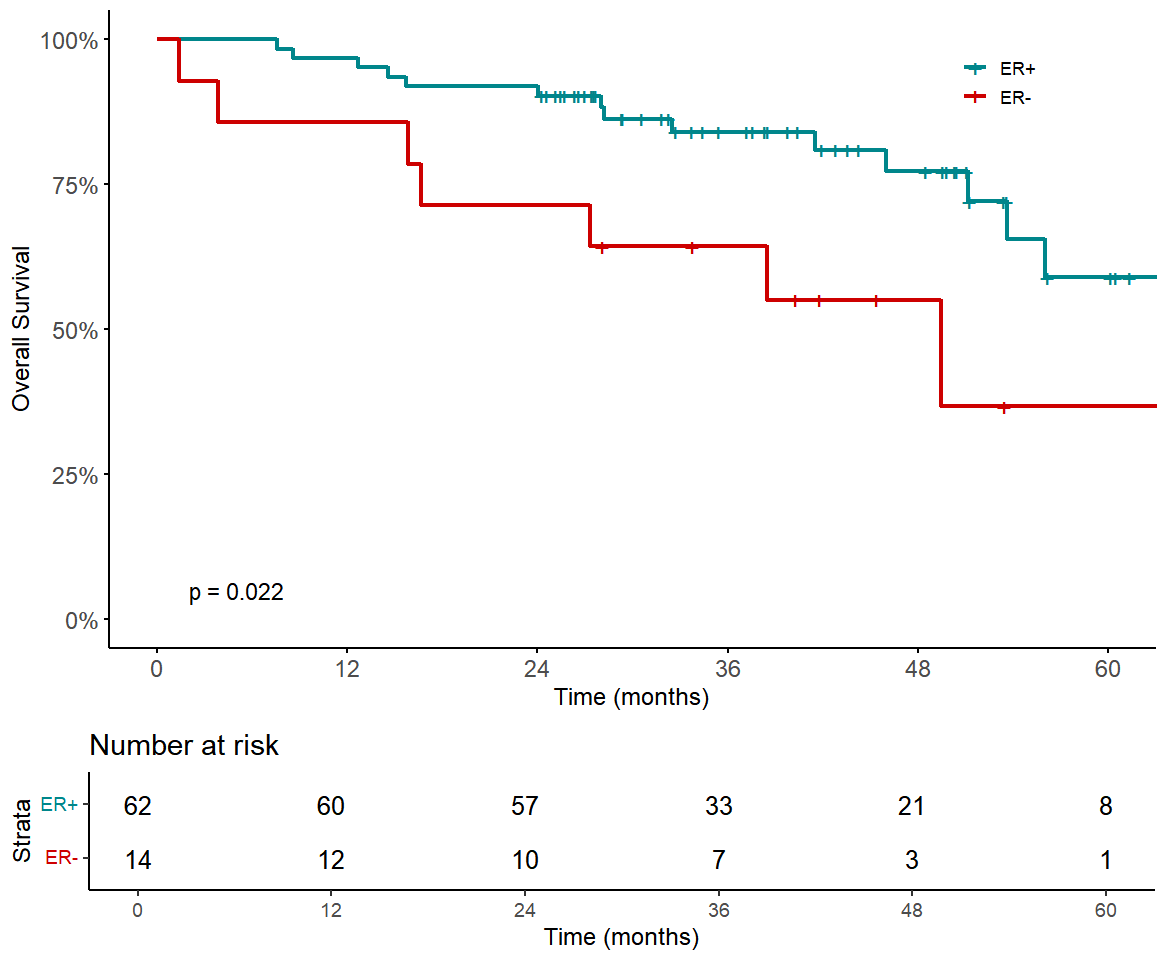
**A**


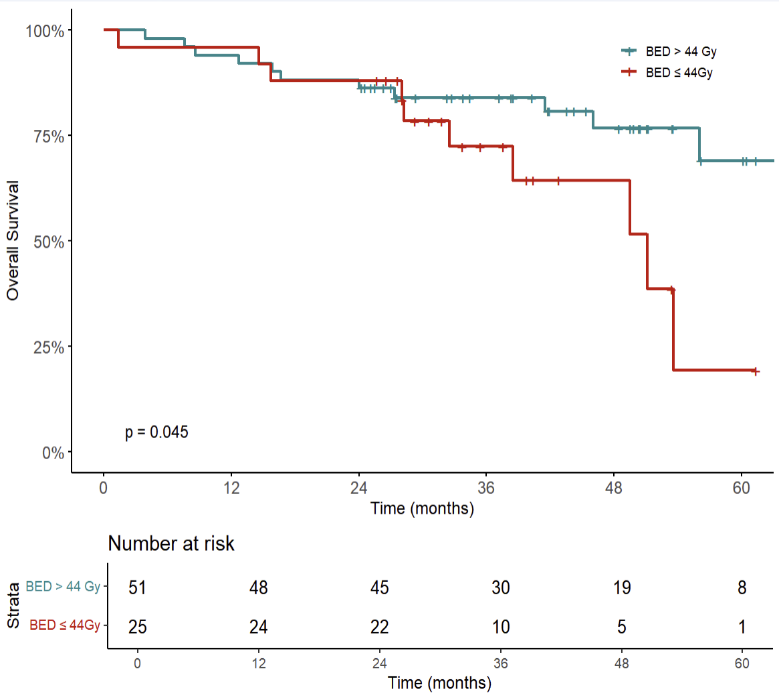
**B**

**C**

**
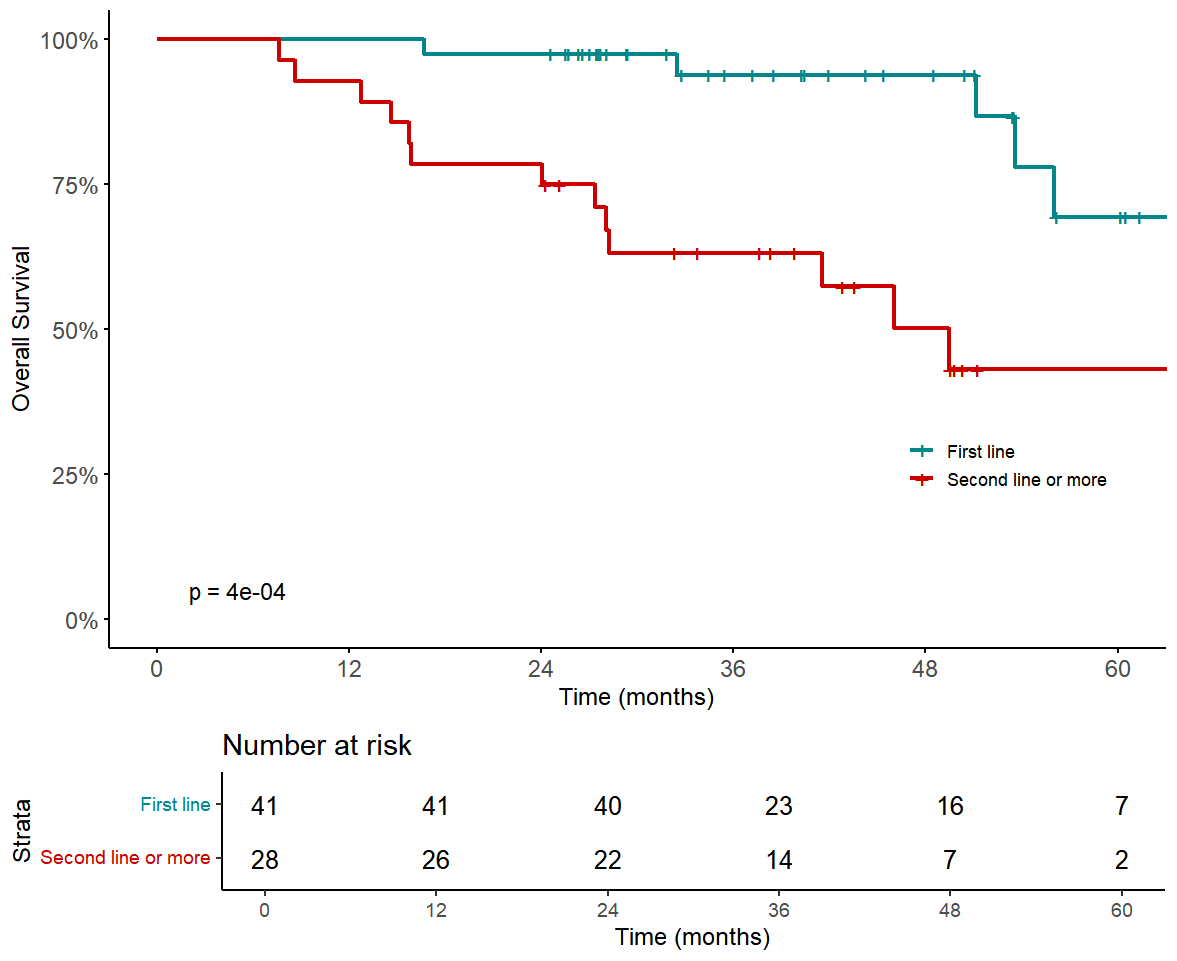
**

**Supplementary Figures S4.** Kaplan-Meier curves of OS according to ER-status (**A**), BED (cut-off: 44 Gy) (**B**) and number of current line (first versus second or more) **(C)**


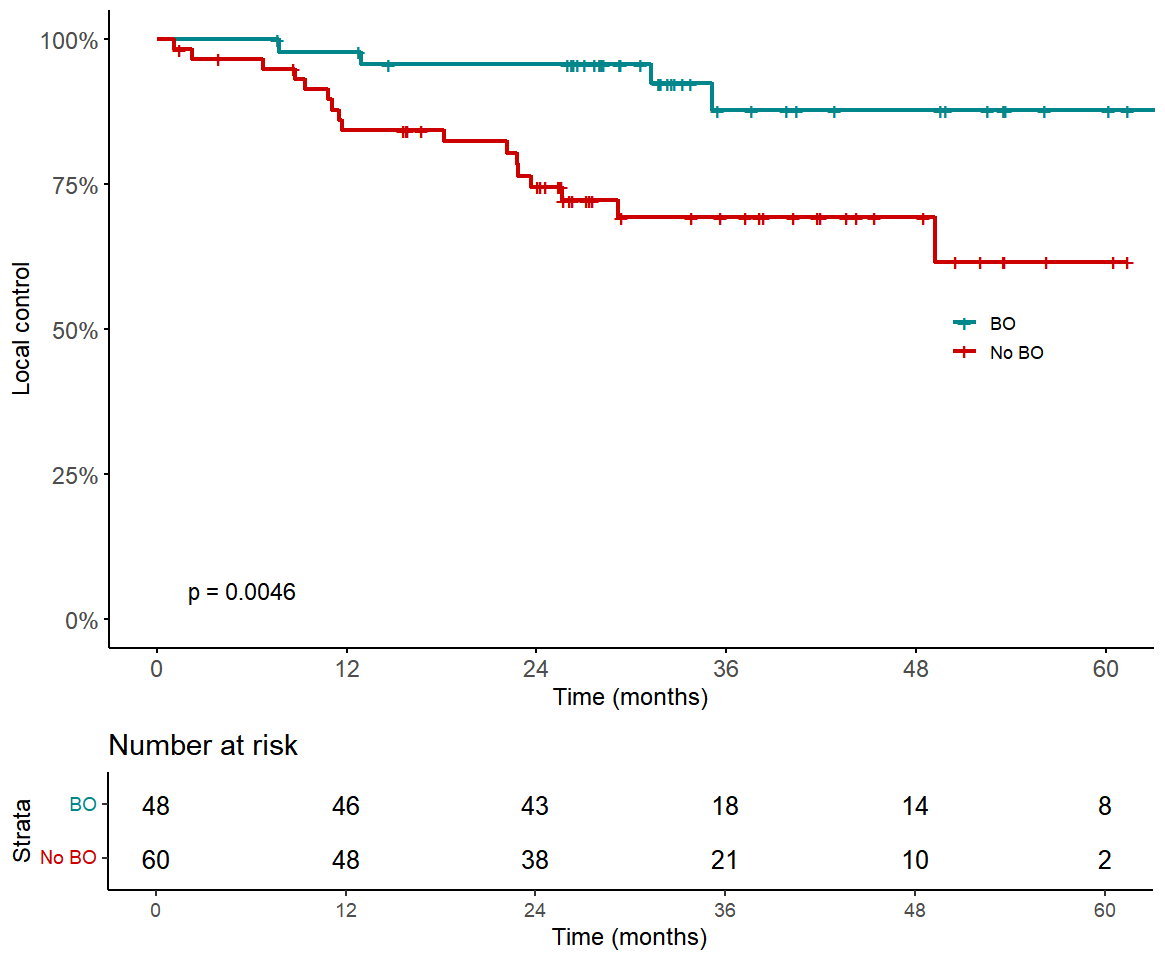
**A**

**
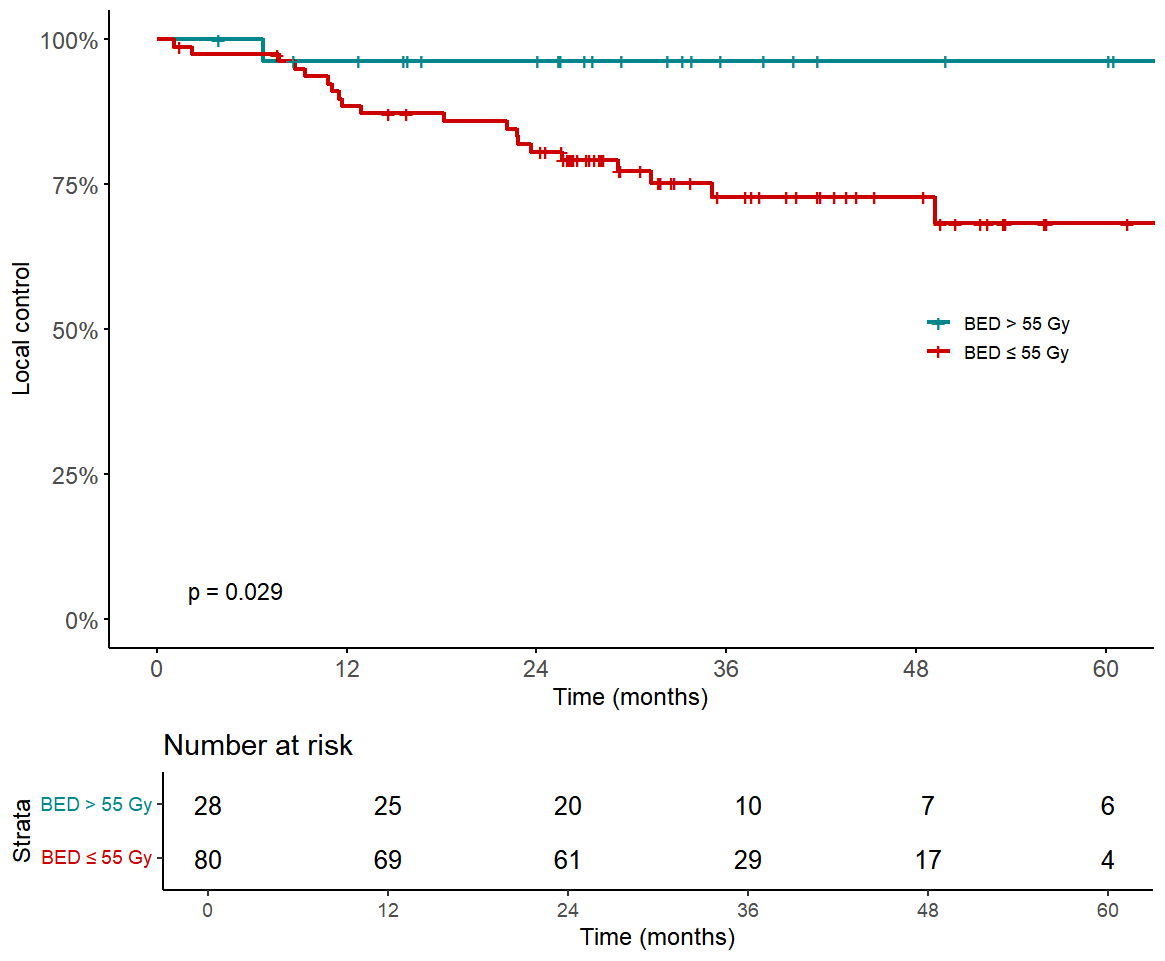
B**

**C**

**
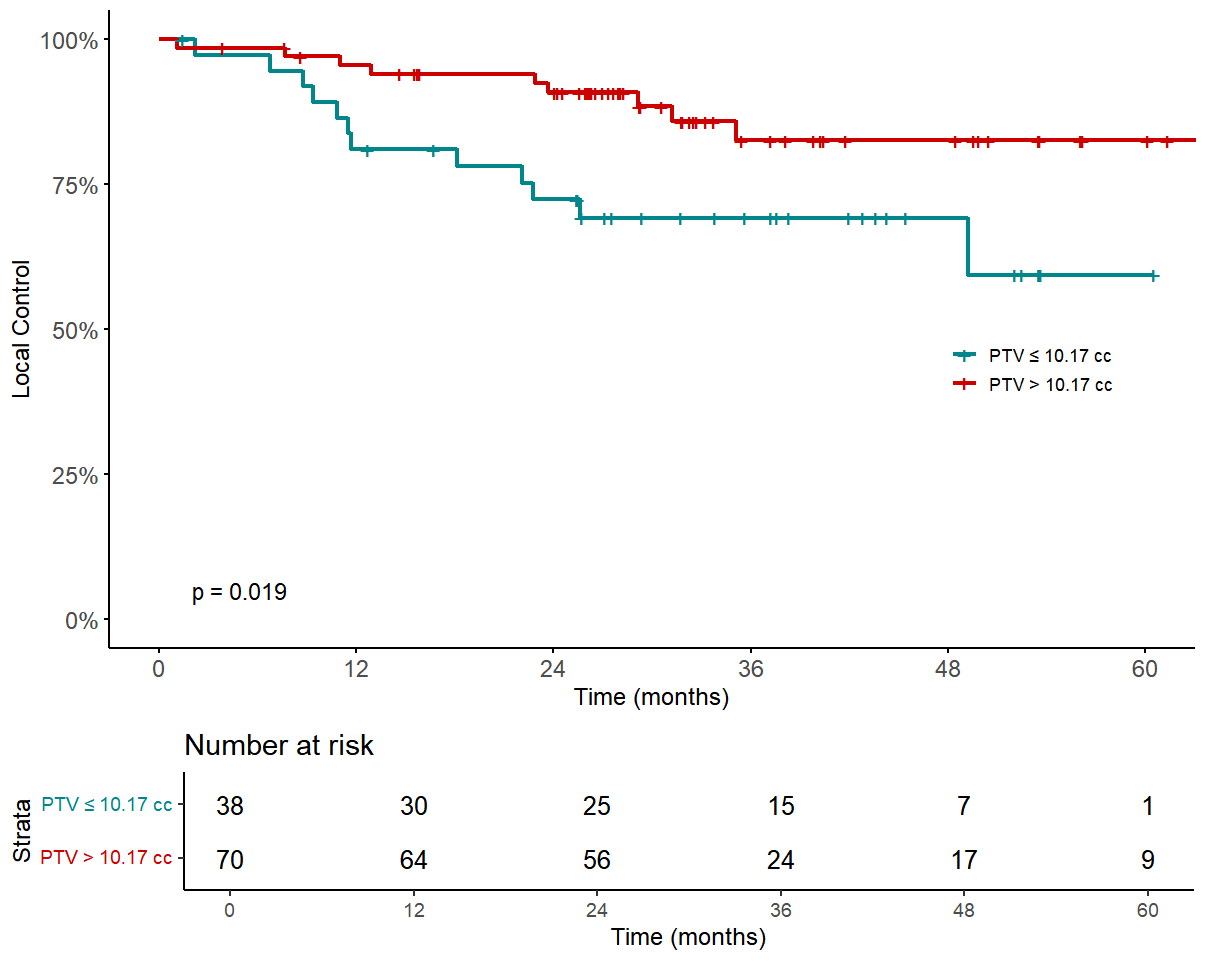
**

**Supplementary Figures S5.** Kaplan-Meier curves of LC according to bone-only metastases status (**A**), BED (cut-off: 55 Gy) (**B**) and PTV size (cut-off: 10.17) (**C**)

**A**


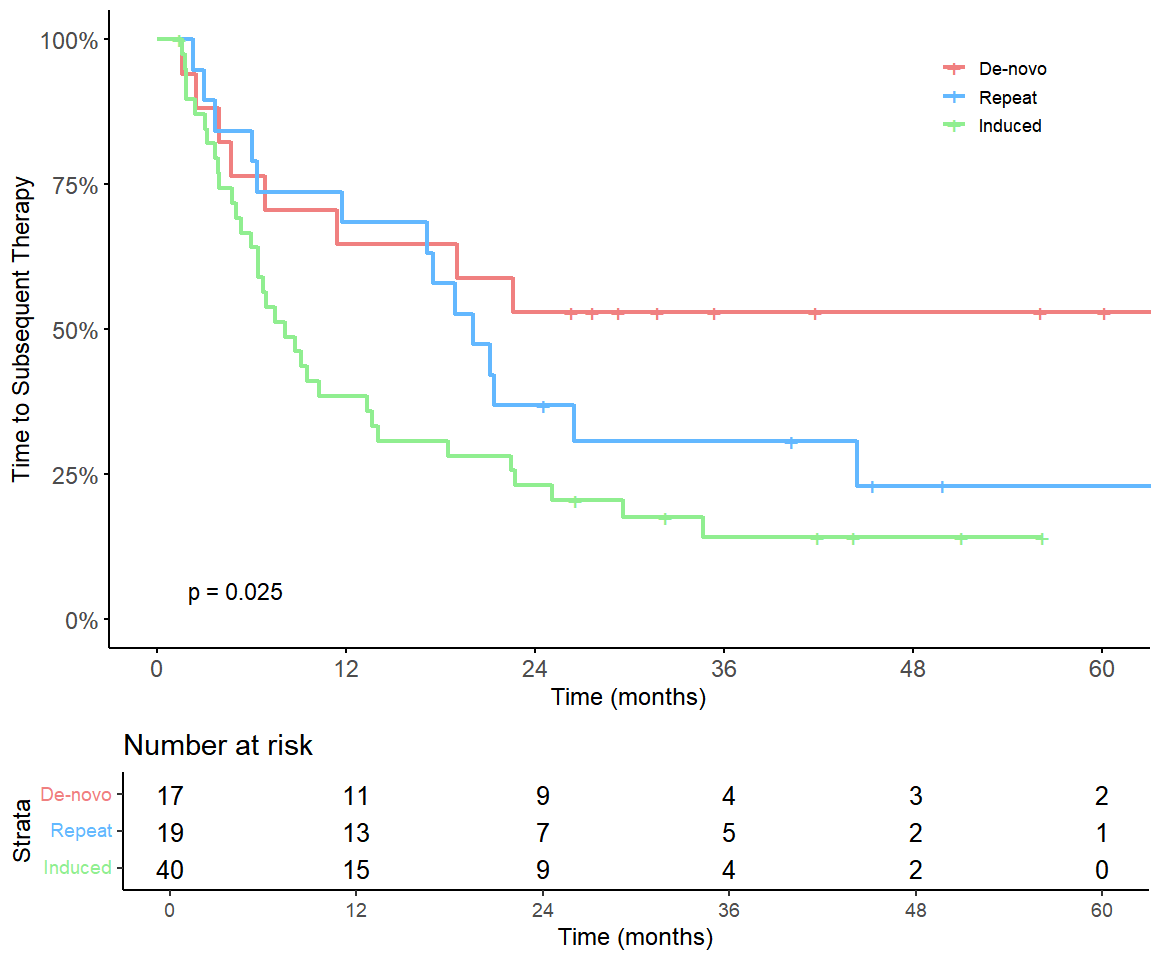


**
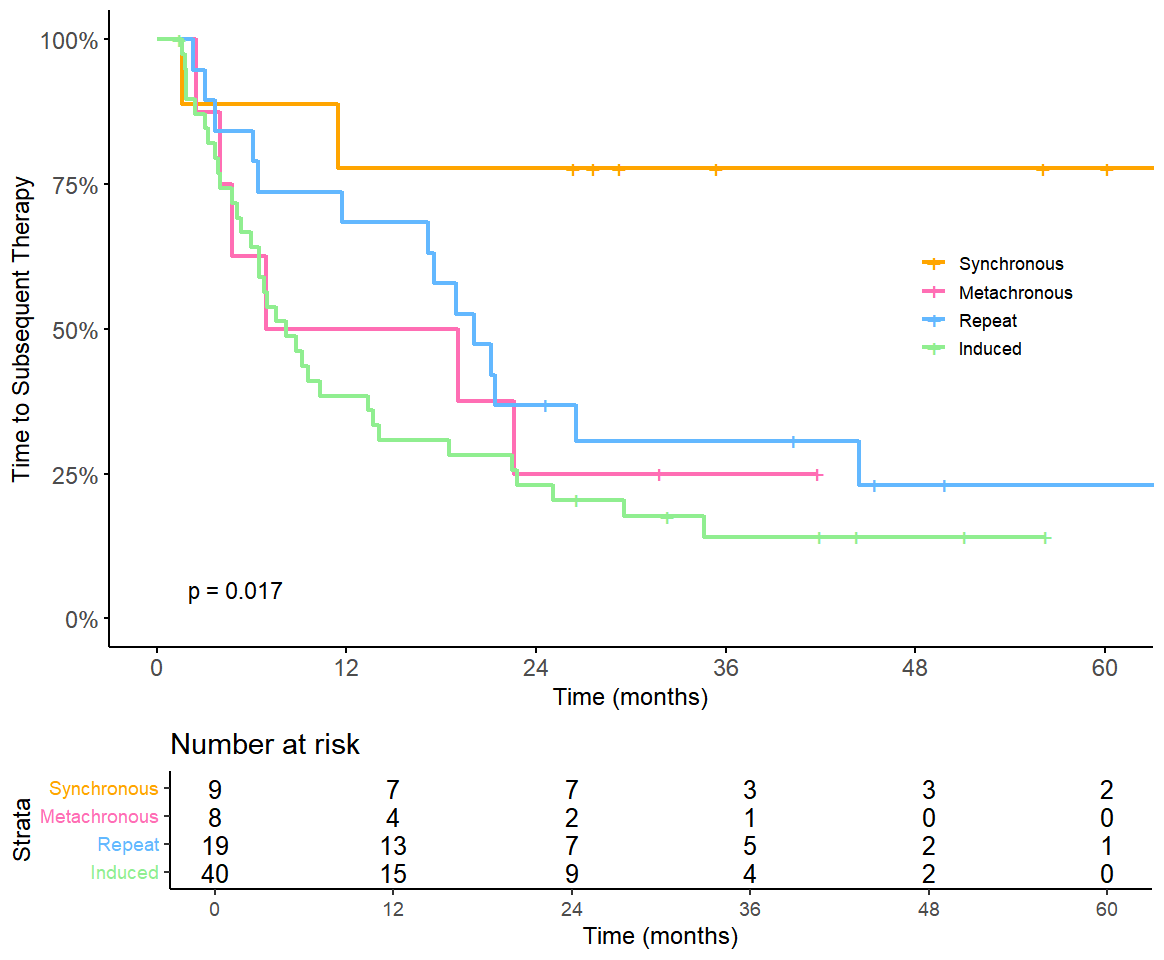
B**


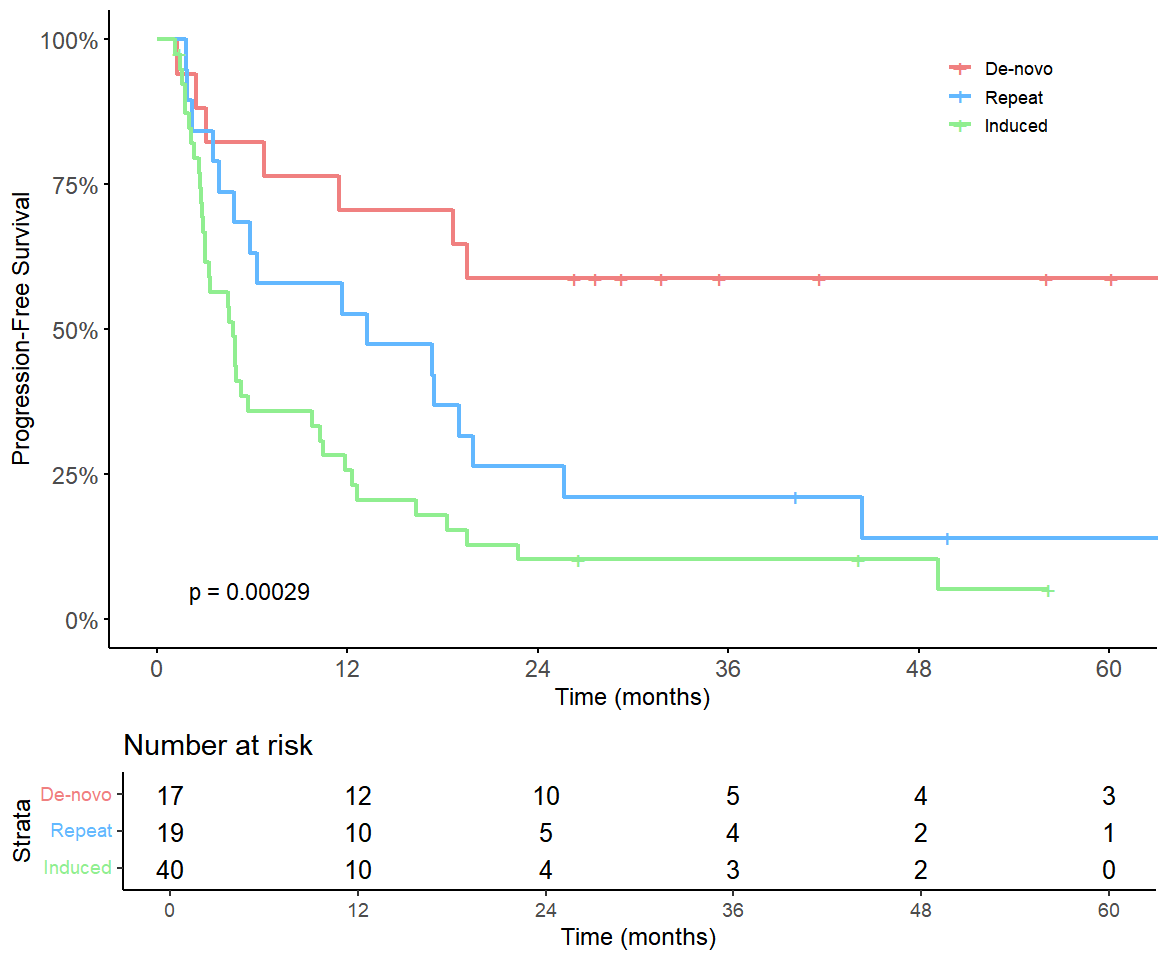
**C**


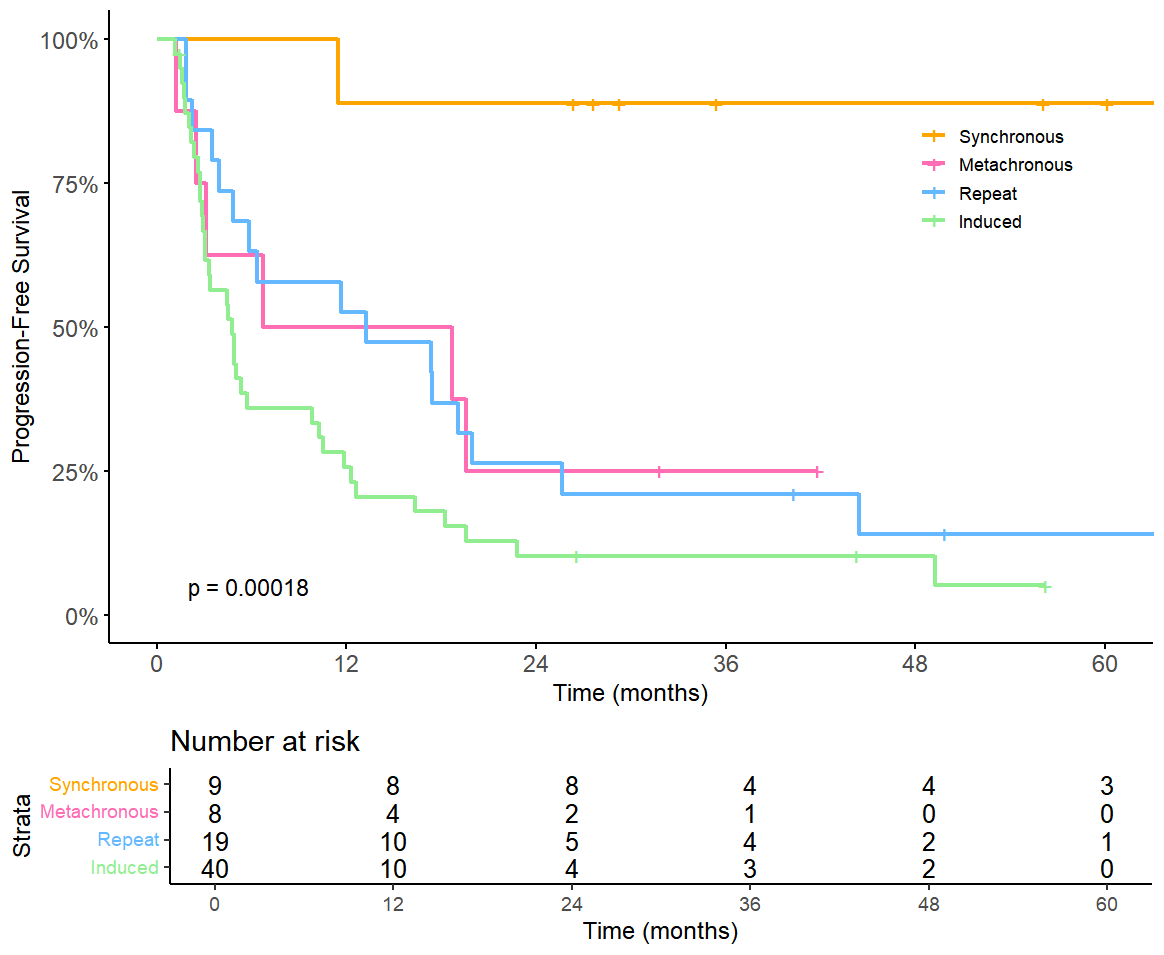
**D**

**
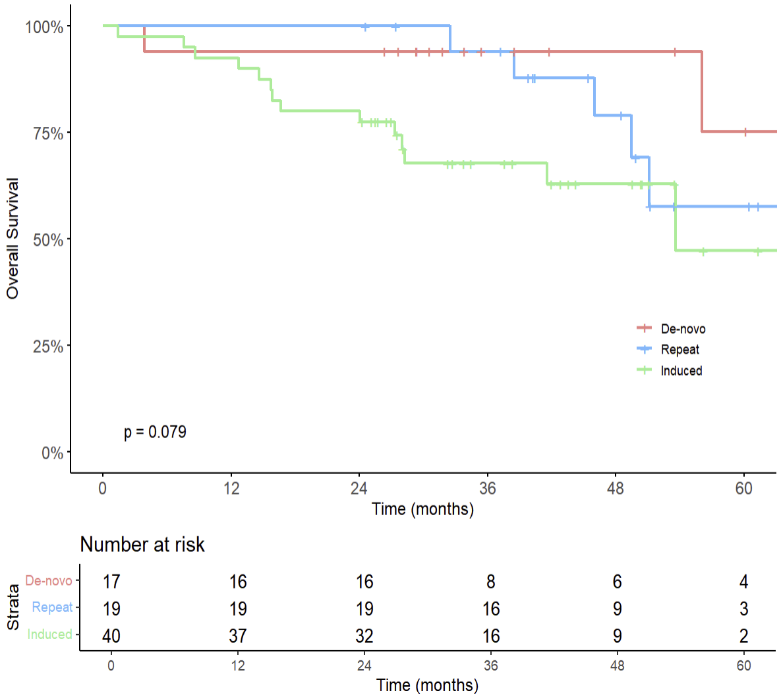
E**


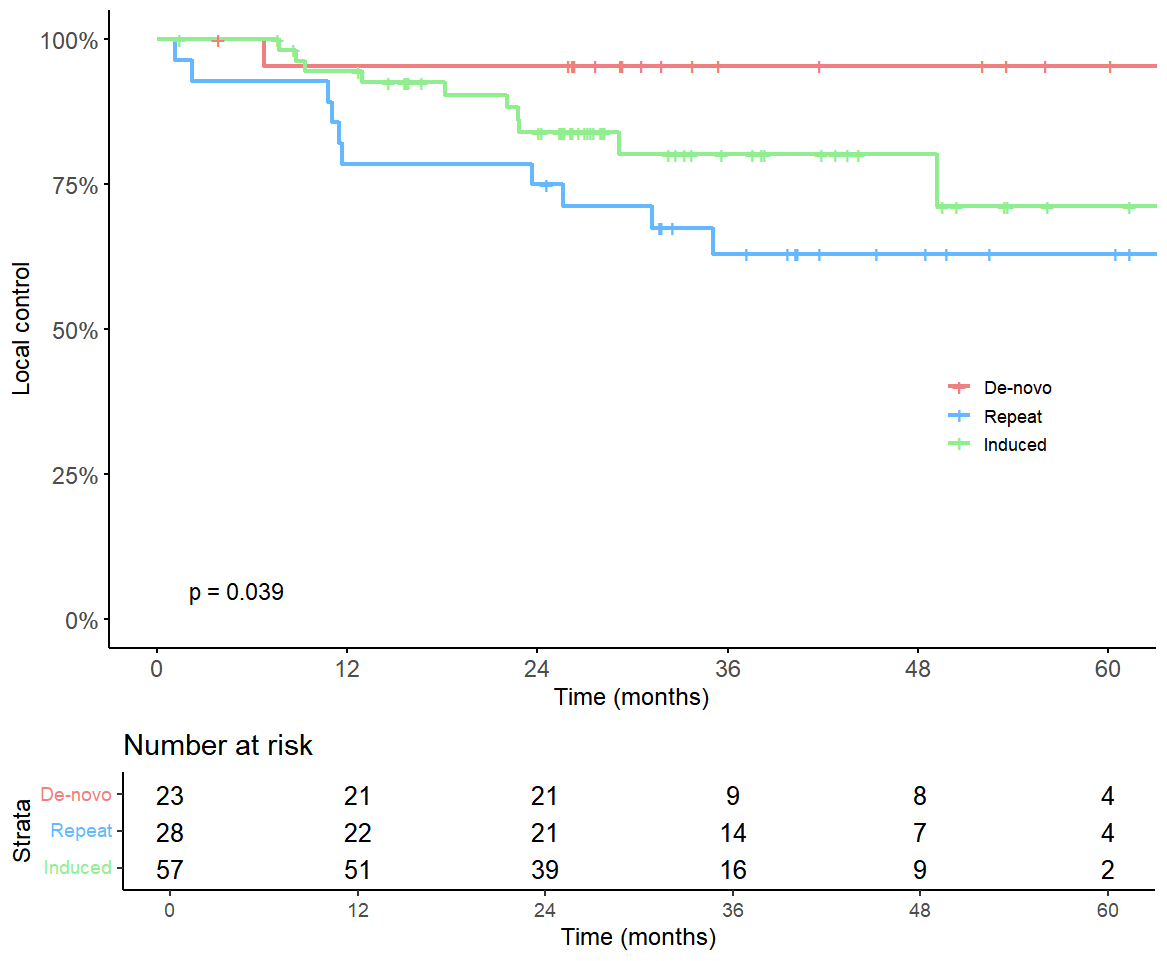
**F**

**Supplementary Figures S6.** Kaplan-Meier curves according to OMD classification

TTST according to de-novo, repeat and induced OMD (**A**) and synchronous, metachronous, repeat and induced OMD (**B**)

PFS according to de-novo, repeat and induced OMD (**C**) and synchronous, metachronous, repeat and induced OMD (**D**)

OS (**E**) and LC (**F**) according to de-novo, repeat and induced OMD and synchronous, metachronous, repeat and induced OMD
